# *CRABP1, C1QL1* and *LCN2* are biomarkers of differentiated thyroid carcinoma, and predict extrathyroidal extension

**DOI:** 10.1186/s12885-017-3948-3

**Published:** 2018-01-10

**Authors:** Ricardo Celestino, Torfinn Nome, Ana Pestana, Andreas M. Hoff, A. Pedro Gonçalves, Luísa Pereira, Bruno Cavadas, Catarina Eloy, Trine Bjøro, Manuel Sobrinho-Simões, Rolf I. Skotheim, Paula Soares

**Affiliations:** 1 i3S - Instituto de Investigação e Inovação em Saúde, Universidade do Porto, Rua Alfredo Allen, 208, 4200-135 Porto, Portugal; 2 IPATIMUP - Institute of Molecular Pathology and Immunology of the University of Porto, Rua Alfredo Allen, 208, 4200-135 Porto, Portugal; 3 Department of Molecular Oncology, Institute for Cancer Research, Norwegian Radium Hospital, Oslo University Hospital, P.O.Box 4953 Nydalen, 0424 Oslo, Norway; 4 School of Allied Health Technologies, Polytechnic of Porto, Rua Dr. António Bernardino de Almeida, 400, 4200-072 Porto, Portugal; 5 Centre for Cancer Biomedicine, Faculty of Medicine, University of Oslo, 0424 Oslo, Norway; 6 ICBAS - Abel Salazar Biomedical Sciences Institute of the University of Porto, 4050-313 Porto, Portugal; 7 IBMC - Instituto de Biologia Molecular e Celular, Universidade do Porto, Rua Alfredo Allen, 208, 4200-135 Porto, Portugal; 8 Department of Medical Biochemistry, Norwegian Radium Hospital, Oslo University Hospital, 0424 Oslo, Norway; 9 Institute of Clinical Medicine, University of Oslo, 0318 Oslo, Norway; 10 Department of Pathology, Medical Faculty, University of Porto, 4200-319 Porto, Portugal; 11 Department of Pathology, Centro Hospitalar de São João, 4200-319 Porto, Portugal

**Keywords:** *CRABP1*, *CILP*, *C1QL1*, *LCN2*, Cancer, Thyroid, Biomarker, TCGA, RNA sequencing, Gene expression

## Abstract

**Background:**

The prognostic variability of thyroid carcinomas has led to the search for accurate biomarkers at the molecular level. Follicular thyroid carcinoma (FTC) is a typical example of differentiated thyroid carcinomas (DTC) in which challenges are faced in the differential diagnosis.

**Methods:**

We used high-throughput paired-end RNA sequencing technology to study four cases of FTC with different degree of capsular invasion: two minimally invasive (mFTC) and two widely invasive FTC (wFTC). We searched by genes differentially expressed between mFTC and wFTC, in an attempt to find biomarkers of thyroid cancer diagnosis and/or progression. Selected biomarkers were validated by real-time quantitative PCR in 137 frozen thyroid samples and in an independent dataset (TCGA), evaluating the diagnostic and the prognostic performance of the candidate biomarkers.

**Results:**

We identified 17 genes significantly differentially expressed between mFTC and wFTC. *C1QL1*, *LCN2*, *CRABP1* and *CILP* were differentially expressed in DTC in comparison with normal thyroid tissues. *LCN2* and *CRABP1* were also differentially expressed in DTC when compared with follicular thyroid adenoma. Additionally, overexpression of *LCN2* and *C1QL1* were found to be independent predictors of extrathyroidal extension in DTC.

**Conclusions:**

We conclude that the underexpression of *CRABP1* and the overexpression of *LCN2* may be useful diagnostic biomarkers in thyroid tumours with questionable malignity, and the overexpression of *LCN2* and *C1QL1* may be useful for prognostic purposes.

**Electronic supplementary material:**

The online version of this article (10.1186/s12885-017-3948-3) contains supplementary material, which is available to authorized users.

## Background

Thyroid cancer is the most frequent type of endocrine cancer with an incidence of 12 cases per 100,000 individuals [1, 2]. More than 95% of thyroid cancer cases originate from follicular epithelial cells [3]. Papillary thyroid carcinoma (PTC) and follicular thyroid carcinoma (FTC) are the most common histotypes. Despite the overall good prognosis of these two main histotypes of differentiated thyroid carcinoma (DTC) [1], some cases progress, develop distant metastases and acquire an unpredictable response to treatment.

The increasing incidence of thyroid cancer has led to the search for good biomarkers that can help in the diagnosis of malignancy and/or predict the clinical behaviour of the tumours. Until now, clinical and histopathological prognostic factors remain the only robust elements to be used for diagnosis and prognosis of patients with thyroid tumours [4], although new markers are revealing some diagnostic or prognostic value per se. As an example, *BRAF* mutations have been shown to be useful for predicting recurrence and/or disease persistence [5], but mostly when associated with other clinicopathological features. Recently, *TERT* promoter mutations revealed an independent prognostic value regarding distant metastasis and survival of patients with thyroid cancer [5].

The classification of benign and malignant thyroid tumours at the histological level still has limitations. Many follicular patterned tumours are typical examples of this difficulty. The classical histological criterion to distinguish FTC from follicular thyroid adenoma (FTA) is the presence of any image of capsular or vascular invasion [6]. This circumstance limits the diagnostic accuracy of fine needle aspiration biopsy (FNAB) in pre-surgical grounds. FTC is subclassified into minimally invasive FTC (mFTC) and widely invasive FTC (wFTC) [3], with the latter having a worse prognosis than mFTC [3, 7, 8]. The molecular mechanisms that orchestrate the invasiveness of FTC cancer cells are poorly understood.

In order to identify molecular alterations associated to thyroid cancer invasion, we used FTC as a model of an encapsulated tumour, studying tumours with different degrees of invasion: two mFTC and two wFTC, using high-throughput paired-end RNA sequencing (RNA-seq) technology. The biomarkers proposed here were validated in a series of DTC, in thyroid cancer cell lines and in the gene expression data from DTC available in The Cancer Genome Atlas (TCGA) [9].

## Methods

### Thyroid cancer samples and cell lines

Two wFTC (cases 1 and 2) and two mFTC (cases 3 and 4) were collected from tumour tissue biobank (Porto). These four tumours from patients with 55–82 years of age were subjected to high-throughput paired-end RNA-seq analyses. Briefly, the patient/case 1 had a tumour measuring 2 cm in diameter and presenting a predominant follicular growth pattern and oncocytic features; patient/case 2 had a tumour measuring 5 cm in diameter and presenting a predominant solid/trabecular growth pattern; patient/case 3 had a tumour measuring 3.7 cm in diameter and presenting a follicular growth pattern; and patient/case 4 had a tumour measuring 4 cm in diameter and presenting a follicular growth pattern.

For comparative and validation purposes, 137 frozen thyroid samples were collected from tumour tissue biobank (Porto): 98 DTC [15 FTC (including the four used in RNAseq), 23 follicular variant of PTC (FVPTC) and 60 PTC], 20 FTA, and 19 normal samples of adjacent/contralateral thyroid tissues from patients with DTC from this series. The histology of all DTC was revised by two pathologists (CE and MSS) and the final classification was made according to the WHO criteria [3]. The clinicopathological features and genetic alterations of the DTC are presented in Table 1, and a briefly description of the series is available in the Additional file 1. Furthermore, ten thyroid cancer cell lines were also used in this study including one cell line derived from one FTC with oncocytic pattern (XTC1), three derived from PTC (BCPAP, K1 and TPC1), and six derived from undifferentiated thyroid carcinoma (C643, HTH74, KAT4, T238, T241, 8505C).

**Table 1:** Clinicopathological and molecular data of patients with differentiated thyroid cancer (DTC) included in the study

	Total	FTC	FVPTC	PTC
Total (%)	98 (100)	15 (15.3)	23 (23.5)	60 (61.2)
Age (n)	98	15	23	60
Mean (years)	43 ± 1.6	53 ± 3.9	43 ± 3.1	40 ± 2.1
Tumour size (n)	93	15	22	56
Mean (cm)	2.8 ± 0.18	3.9 ± 0.46	2.4 ± 0.30	2.7 ± 0.24
Gender (n)	98	15	23	60
Female (%)	84 (85.7)	12 (80)	19 (82.6)	53 (88.3)
Male (%)	14 (14.3)	3 (20)	4 (17.4)	7 (11.7)
Capsule (n)	88	15	21	52
Positive (%)	45 (51.1)	15 (100)	9 (42.9)	21 (40.4)
Capsular invasion (n)	42	15	8	19
Positive (%)	27 (64.3)	15 (100)	2 (25.0)	10 (52.6)
Vascular invasion (n)	89	15	21	53
Positive (%)	48 (53.9)	11 (73)	5 (23.8)	32 (60.4)
Lymph node metastasis (n)	93	15	21	57
Positive (%)	24 (25.8)	–	4 (19.0)	20 (35.1)
Extrathyroidal extension (n)	88	15	21	52
Positive (%)	27 (30.7)	1 (6.67)	2 (9.52)	24 (46.2)
Distant metastasis (n)	93	15	21	57
Positive (%)	5 (5.38)	1 (6.67)	–	4 (7.02)
Lymphocytic thyroiditis (n)	91	15	22	54
Positive (%)	40 (44.0)	4 (26.7)	8 (36.4)	28 (51.9)
Oncocytic (n)	89	15	21	53
Positive (%)	17 (19.1)	6 (40.0)	–	11 (20.8)
*PAX8-PPARG* rearrangements (n)	98	15	23	60
Positive (%)	2 (2.04)	1 (6.67)	1 (4.35)	–
*RET/PTC* rearrangements (n)	98	15	23	60
Positive (%)	17 (17.3)	1 (6.67)	3 (13.0)	13 (21.7)
*RET/PTC1* rearrangement (n)	98	15	23	60
Positive (%)	12 (12.2)	1 (6.67)	1 (4.35)	10 (16.7)
*RET/PTC3* rearrangement (n)	98	15	23	60
Positive (%)	2 (2.04)	–	1 (4.35)	1 (1.67)
*BRAF* mutation (n)	98	15	23	60
Positive (%)	21 (21.4)	–	2 (8.70)	19 (31.7)
*NRAS* mutation (n)	98	15	23	60
Positive (%)	16 (16.3)	5 (33.3)	5 (21.7)	6 (10.0)
*TERT* promoter mutation (n)	98	15	23	60
Positive (%)	3 (3.06)	2 (13.3)	1 (4.35)	–

n, number of cases with available data

FTC, follicular thyroid carcinoma; FVPTC, follicular variant of papillary thyroid carcinoma; PTC, papillary thyroid carcinoma

### High-throughput paired-end RNA-seq

Library construction was performed using the TruSeq RNA Sample Prep Kit v2 according to protocol (Illumina Inc., San Diego, CA, USA), including poly-A mRNA isolation, fragmentation, and gel-based size selection. Shearing to about 250 bp fragments was achieved using the Covaris S2 focused-ultrasonicator (Covaris Inc., Woburn, MA, USA). Sequencing was performed according to the paired-end RNA-seq protocols from Illumina for Solexa sequencing on a Genome Analyzer IIx with paired-end module (Illumina Inc). Seventy-six bps were sequenced, from each side of a fragment of about 250 bp long.

A particular attention was paid to genes differentially expressed between mFTC and wFTC. Reads marked by the Illumina pipeline (Bustard.py, OLB 1.6.0 and 1.8.0) as *passed filtering* were used in the analysis.

Gene expression levels in FTCs were computed by using Cufflinks v1.1.0 [10] with the Illumina iGenomes Ensembl GRCh37 data set (2011–06-20) as reference, on reads aligned with TopHat v1.3.3 [11]. Gene expression levels were compared between the two wFTC (case 1 and 2) and two mFTC (case 3 and 4). Results were represented as fold change of gene expression in wFTC comparing with mFTC. Values of fold change in gene expression were at logarithmic scale (log2). Genes and transcripts were considered differentially expressed between mFTC and wFTC whenever *Q* value <0.05.

### Isolation of nucleic acids, reverse-transcription PCR and cDNA sanger sequencing

Frozen tissue samples available from thyroid tumours (*n* = 118) and adjacent/collateral normal thyroid tissues (*n* = 19) were crushed and homogenized. Total RNA from tissues and thyroid cancer derived cell lines was extracted using TRIzol® Reagent (Ambion®, Life Technologies™, CA, USA), according to the manufacturer’s protocol. Genomic DNA was extracted using the Genomic DNA Purification Kit (Citomed, Lisbon, Portugal) according to the manufacturer’s protocol.

cDNA was synthesized from 1 μg of RNA at 42 °C for 60 min, using oligo (dT) primers and M-MuLV reverse transcriptase (Fermentas, Thermo Scientific, St. Leon-Rot, Germany). The reverse-transcription PCR (RT-PCR) products were analysed by cDNA Sanger sequencing using the Big Dye terminator version 3.1 cycle (Applied Biosystems, Foster City, CA, USA). The samples were analysed in an automated sequencing machine (ABI Prism 3100 Genetic Analyser, Applied Biosystems, Foster City, CA, USA).

### Screening for *PAX8/PPARG*, *RET/PTC1* and *RET/PTC3* rearrangements, *BRAF* and *NRAS* mutations, and *TERT* promoter mutations in thyroid cancer series

The 98 DTC were further characterized for selected molecular alterations known to be common in thyroid tumours. PCR followed by Sanger sequencing was used on genomic DNA for detecting mutations in the most frequent hotspot regions of *BRAF* (exon 15), *NRAS* (exon 2), and promoter region of *TERT*, according to previously described procedures [12, 13]. In our previous works we verified that *HRAS* and *KRAS* mutations were very rare (or absent) events. For that reason, we only screened mutations for NRAS gene.

The presence of *PAX8*-*PPARG* and *RET/PTC* rearrangements were determined by RT-PCR followed by Sanger sequencing of PCR products. Some positive cases were confirmed by fluorescence in situ hybridisation (FISH), following the procedures described previously [12].

### Real-time quantitative reverse transcription PCR

Real-time quantitative PCR (qPCR) for *C1QL1*, *LCN2*, *IL22RA1*, *MAMDC2*, *CILP*, *ASXL3*, *CRABP1*, and *SCUBE3* genes was performed in the two wFTC (cases 1 and 2) and two mFTC (cases 3 and 4) used in paired-end RNA-seq, and their corresponding normal thyroid tissue. qPCR for *C1QL1*, *LCN2*, *CILP*, and *CRABP1* were also performed in the remaining series of DTC, in FTA and in normal thyroid tissues, as well as in thyroid cancer cell lines.

cDNA was amplified for the *C1QL1*, *LCN2*, *IL22RA1*, *MAMDC2*, *CILP*, *ASXL3*, *CRABP1*, and *SCUBE3* by qPCR using SYBR® Green PCR Master Mix (Applied Biosystems, Foster City, CA, USA). A qPCR assay targeting Hypoxanthine phosphoribosyltransferase 1 (*HPRT*) was used as the housekeeping control. Primer sequences are available on request. Expression levels were obtained as the average cycle threshold (CT) of at least two replicas. A CT = 35 was assigned for target genes that were not expressed in samples with positive expression of the housekeeping control gene (*HPRT)*.

The relative quantification of target genes was determined using the comparative CT method (2^-ΔΔCT^) which was previously validated by Livak’s Linear Regression Method (Sequence Detector User Bulletin 2; Applied Biosystems) [14]. This provided the fold changes (2^-ΔΔCT^) in gene expression normalized to an internal control gene (*HPRT*), and relative to one pool of normal thyroid tissues (calibrator). Expression values (2^-ΔΔCT^) were normalized at logarithmic scale (log2). For *C1QL1* and *LCN2* genes, fold change [log2 (2^-ΔΔCT^)] > 1 was considered as gain of expression, and fold change [log2 (2^-ΔΔCT^)] ≤ 1 was established as normal gene expression. For *CRABP1* and *CILP* genes, fold change [log2 (2^-ΔΔCT^)] < −1 was considered as loss of expression, and fold change [log2 (2^-ΔΔCT^)] ≥ −1 was established as normal gene expression. The diagnostic performance (sensitivity and specificity) of those cut-off values are presented in Table 2.

**Table 2:** Diagnostic performance of potential biomarkers for differentiated thyroid cancer (DTC)

Biomarker	Cut-off	Sensitivity (%)	Specificity (%)	
		DTC	DTC vs normal thyroid	DTC vs FTA
*C1QL1*	>1	62.9 (52.0–72.9)	82.4 (56.6–96.0)	58.8 (33.0–81.5)
*LCN2*	>1	64.0 (52.9–74.0)	73.3 (44.9–92.1)	83.3 (58.6–96.2)
*CRABP1*	<−1	84.3 (75.0–91.1)	88.9 (65.3–98.3)	35.7 (12.9–64.8)
*CILP*	<−1	61.4 (50.4–71.6)	66.7 (35.0–89.9)	53.0 (27.9–77.0)

Sensitivities and specificities are indicated comparing DTC with normal thyroid tissue or follicular thyroid adenoma (FTA)

### Data from the cancer genomic atlas (TCGA)

RNASeq (version v2) information were extracted from TCGA [15] for 393 DTC and 59 thyroid normal samples, for *C1QL1*, *LCN2*, *CRABP1* and *CILP* genes. Gene expression values for each sample are normalized read counts, estimated by using RSEM software [16].

### Statistical analysis

Statistical analysis was conducted with SPSS version 22.0 (SPSS Inc., Chicago, IL, USA). The results are expressed as percentage or mean ± SE. Statistical analysis was performed both on the whole DTC series and on the different subgroups: FTC, FVPTC and PTC. Receiver Operating Characteristics (ROC) curves for individual biomarkers were generated using log2 (2^-ΔΔCT^) gene expression values and thyroid tissue type (DTC against normal or FTA) as input. For evaluation of the combined biomarker panel, the sum of log2 (2^-ΔΔCT^) expression values from genes with gain (*C1QL1* and *LCN2*) and loss (*CRABP1* and *CILP*) in DTC were used. Spearman’s rho test (non-parametric) was used for evaluating the correlation of the expressions between different genes. Fisher’s exact test, t-test (unpaired, two-tailed), Mann–Whitney U test, and ANOVA were used when appropriate. The predictive value of *C1QL1*, *LCN2*, *CRABP1* and *CILP* expression as a prognostic factor in thyroid cancer and their correlation with clinicopathological factors – age, tumour size, and extrathyroidal extension were assessed using univariate and multivariate logistic regression models. Test results with *P-*values <0.05 were considered statistically significant.

## Results

### Differential gene expression in mFTC and wFTC

**Fig. 1:**
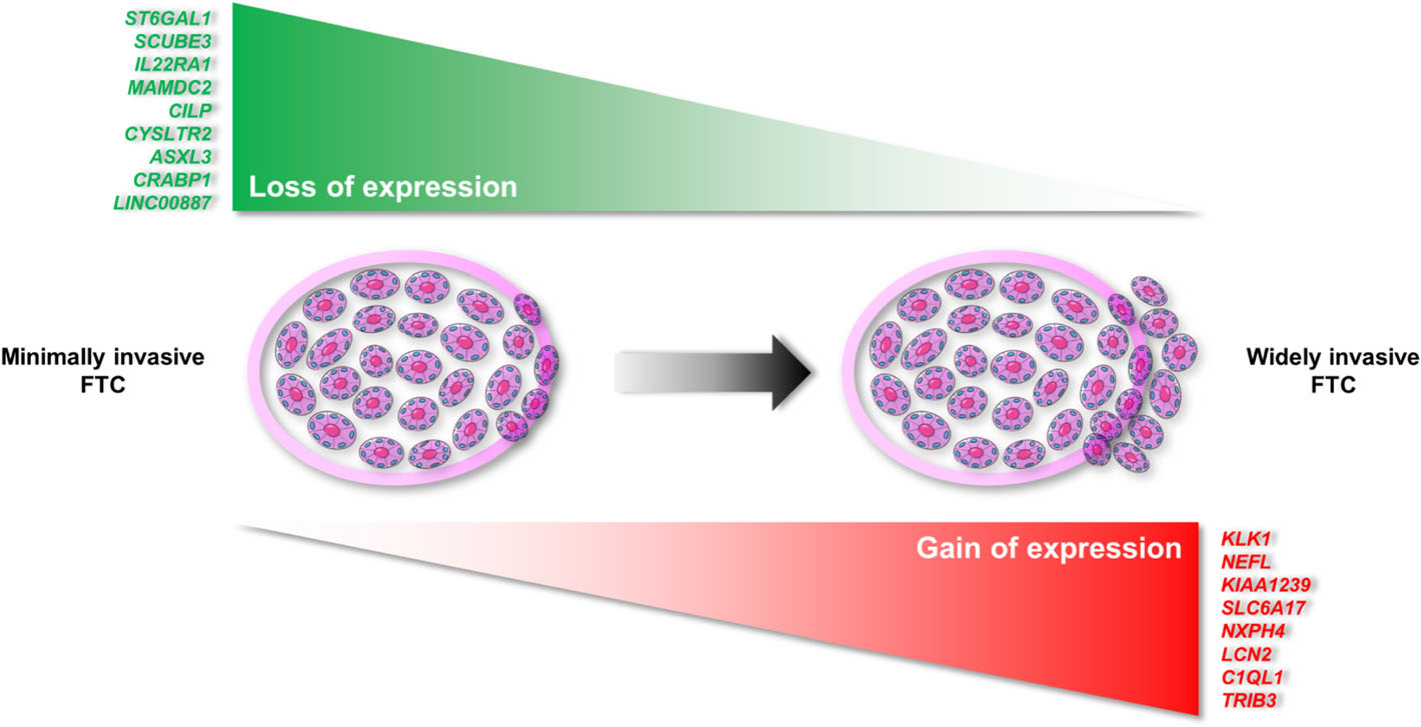
Differentially expressed genes found between minimally and widely invasive follicular thyroid carcinoma (FTC). Gene expression was measured by high-throughput paired-end RNA sequencing and using statistically significant values (*Q* < 0.05). Genes with lower expression in widely invasive FTC as compared with minimally invasive FTC are listed at the top of the figure (green). Genes with higher expression in widely invasive FTC as compared with minimally invasive FTC are listed at the bottom of the figure (red). Details of genes, expression levels, statistical values, and molecular function of the genes are found in Additional file 2: Table S1 for differentially expressed genes and in Additional file 3: Table S2 for differentially expressed transcripts

Differential expression analysis of the paired-end RNA-seq data identified 17 genes with significantly differential expression between mFTC and wFTC (Fig. 1, Additional file 2: Table S1 and Additional file 3: Table S2). Increased gene expression of *KLK1*, *NEFL*, *KIAA1239*, *SLC6A17*, *NXPH4*, *LCN2*, *C1QL1* and *TRIB3*, and decreased gene expression of *ST6GAL1*, *SCUBE3*, *IL22RA1*, *MAMDC2*, *CILP*, *CYSLTR2*, *ASXL3*, *CRABP1* and *LINC00887* were observed in wFTC in comparison with mFTC. *C1QL1*, *LCN2*, *IL22RA1*, *MAMDC2*, *CILP*, *ASXL3*, *CRABP1*, and *SCUBE3* genes were selected for validation through a customized filtering based on gene expression in thyroid tumours and normal thyroid tissues datasets [17–19], assessed from Oncomine™ and in the literature available. Validation of expression levels was done in the four FTCs from paired-end RNA-seq and in their matched normal tissue by qPCR. Gain of *C1QL1* and *LCN2* expression, and loss of *IL22RA1*, *MAMDC2*, *CILP*, *ASXL3*, *CRABP1*, and *SCUBE3* expression were confirmed in wFTC compared with mFTC and between the four FTC and their respective corresponding normal thyroid tissue (Additional file 4: figure S1). Based on the accuracy of gene expression levels obtained from paired-end RNA-seq and qPCR in the FTC and in the gene expression levels in their normal thyroid tissues by qPCR, *C1QL1*, *LCN2*, *CRABP1* and *CILP* genes were selected to be tested as biomarkers in a well-characterized series of DTC (Table 1).

Additionally, we identified and experimentally verified a set of 21 fusion transcripts expressed by DTC (Additional file 5: figure S2; Additional file 6: Table S3; Additional file 7: Table S4). However, the fusion transcripts are not likely to be biomarkers because they were also detected in normal thyroid tissues. Further details can be found in the Additional file 8.

### *C1QL1*, *LCN2*, *CRABP1*, and *CILP* as biomarkers in DTC

A series of 98 DTC (15 FTC, 23 FVPTC and 60 PTC) and a pool of 19 normal thyroid tissues and 20 FTA were used to evaluate the performance of *C1QL1*, *LCN2*, *CRABP1*, and *CILP* as biomarkers of DTC. Measurements of the expression levels of those genes were done by qPCR. *C1QL1*, *LCN2*, *CRABP1* and *CILP* were differentially expressed in DTC in comparison with normal thyroid tissues (Additional file 9: figure S3), with statistical significance (*P* = 0.002, *P* = 0.005, *P* < 0.001 and *P* = 0.018, respectively). Notably, such significant differences were also observed for *LCN2* and *CRABP1* expression when comparing DTC against FTA (*P* < 0.001 and *P* = 0.002, respectively), and FTA against normal thyroid tissues (*P* = 0.013 and *P* = 0.022, respectively).

**Fig. 2:**
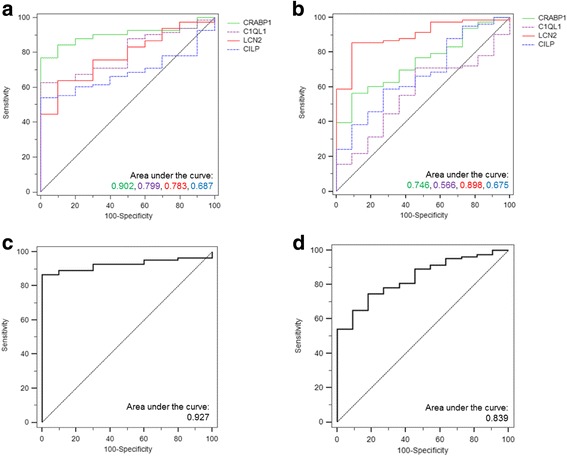
Receiver Operating Characteristics (ROC) curves for gene expression of potential biomarkers in thyroid cancers. ROC curves for gene expression of individual and combined potential biomarkers in differentiated thyroid cancers (DTC) versus normal thyroid tissues and DTC versus FTA. The area under the ROC curve (AUC) represents the accuracy of the individual and combined potential biomarkers for distinguishing differentiated thyroid cancers (DTC) from normal thyroid tissue samples or follicular thyroid adenoma (FTA) (**a-d**). DTC versus normal thyroid for individual (**a**) and combined (**c**) potential biomarkers with gain (*C1QL1*and *LCN2*) and loss (*CRABP1* and *CILP*) of gene expression in cancer. DTC versus FTA for individual (**b**) and combined (**d**) potential biomarkers with gain (*C1QL1* and *LCN2*) and loss (*CRABP1* and *CILP*) of gene expression in cancer. Asymptotic significance, standard error and 95% confidence interval measurements for all values can be found in Additional file 10: Table S5

In order to test the aforementioned biomarkers as putative diagnostic tools for thyroid cancer, ROC curves were performed (Fig. 2, Additional file 10: Table S5). In the ROC analysis, comparing DTC versus normal thyroid (Fig. 2a), the areas under of the ROC curve (AUC) for *C1QL1*, *LCN2*, *CRABP1* and *CILP* were 0.799 (*P* = 2.1E-3), 0.783 (*P* = 3.6E-3), 0.902 (*P* = 3.4E-5), and 0.687 (*P* = 5.5E-2), respectively. When these potential biomarkers were combined in a panel of gene expression, the AUC value was 0.927 (*P* = 1.1E-5) in DTC versus normal thyroid (Fig. 2c), providing a slight improvement over the AUC results obtained in each individual gene. Comparing DTC with FTA, the AUC values for *C1QL1*, *LCN2*, *CRABP1* and *CILP* were 0.566 (*P* = 4.8E-1), 0.898 (*P* = 1.9E-5), 0.746 (*P* = 8.3E-3), and 0.675 (*P* = 6.1E-2), respectively (Fig. 2b). When these potential biomarkers were combined in a panel of gene expression, the AUC value was 0.839 (*P* = 2.7E-4) in DTC versus FTA (Fig. 2d), not improving the result in comparison with those of the AUC of *LCN2* gene. *CRABP1* was the best biomarker for the detection of DTC when tested against normal thyroid. On the other hand, *LCN2* was the best biomarker for the detection of DTC when tested against FTA.

We also tested gene expression levels from an additional series of 393 DTC and 59 normal thyroid tissues available in TCGA [15]. *C1QL1*, *LCN2* and *CRABP1* were differentially expressed in DTC in comparison with normal thyroid tissues (Additional file 11: figure S4), with statistical significance (*P* < 0.001).

### Clinical and molecular associations with gain of *C1QL1* expression

**Fig. 3:**
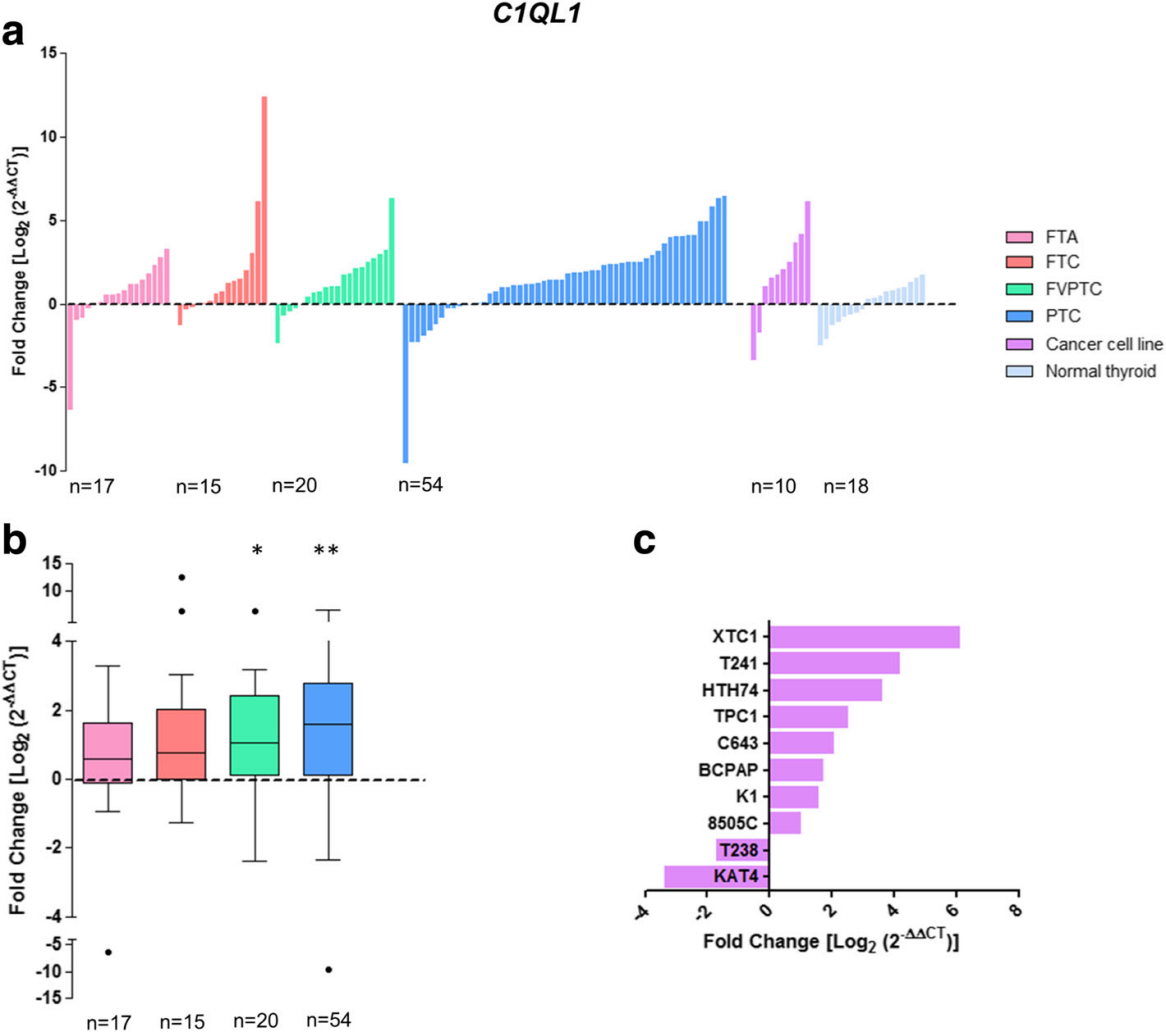
Differential gene expression of *C1QL1* in thyroid tumours and normal tissues. Gene expression was measured in follicular thyroid adenoma (FTA), follicular thyroid cancer (FTC), follicular variant of papillary thyroid carcinoma (FVPTC), papillary thyroid carcinoma (PTC), normal thyroid tissues and thyroid cancer cell lines by real-time quantitative PCR. Histogram showing the gene expression of each sample ordered by expression levels (**a**). Box plot representation (median and Tukey whiskers) showing gene expression of each subgroup of thyroid tumours – FTA, FTC, FVPTC and PTC (**b**). Gene expression of the thyroid cancer cell lines (**c**). Gene expression was calibrated by the pool of normal thyroid tissues. Statistically significant values: *P = 0.015, **P = 0.001

*C1QL1* expression levels were successful measured by qPCR in 89 DTC (15 FTC, 20 FVPTC and 54 PTC), 10 thyroid cancer cell lines, 17 FTA, and 18 matched thyroid normal tissues (Fig. 3a). Gain of *C1QL1* expression in relation to thyroid normal tissues was found in FTC, FVPTC and PTC (Fig. 3b), but the differences only achieved the threshold of statistical significance in FVPTC (*P* = 0.015) and PTC (*P* = 0.001). Additionally, eight out of ten (80%) thyroid cancer cell lines showed gain of *C1QL1* expression (Fig. 3c). Gain of *C1QL1* expression was significantly associated with extrathyroidal extension (*P* = 0.003) and lymphocytic thyroiditis (*P* = 0.003) in the DTC samples (Table 3). All the cases with *BRAF* mutations present gain of *C1QL1* expression (*P* < 0.001). In FTC, gain of *C1QL1* expression was present in 71% of wFTC and absent in mFTC (*P* = 0.007; Additional file 12: Table S6), and lymphocytic thyroiditis was only present in FTC with gain of *C1QL1* expression (*P* = 0.026). In FVPTC, absence of *NRAS* mutations was significantly associated to the gain of *C1QL1* expression (*P* = 0.014; Additional file 13: Table S7). PTC with *C1QL1* gain were significantly larger (2.96 ± 0.33 cm) than PTC without gain of expression (1.78 ± 0.25 cm; *P* = 0.021; Additional file 14: Table S8). Gain of *C1QL1* expression in PTC was also significantly associated with the presence of extrathyroidal extension (*P* = 0.006), and *BRAF* mutations (*P* = 0.001).

**Table 3:** Clinicopathological and genetic data of differentiated thyroid cancers classified by classes based on gene expression

	*C1QL1* fold change			*LCN2* fold change			*CRABP1* fold change			*CILP* fold change		
	Normal ≤ 1	Gain > 1	*P* value	Normal ≤ 1	Gain > 1	*P* value	Loss < −1	Normal ≥ −1	*P* value	Loss < −1	Normal ≥ −1	*P* value
DTC (*n* = 98)												
Age (n)	33	56	NS (0.318)	31	55	NS (0.614)	75	14	NS (0.826)	54	34	NS (0.474)
Mean (years)	40.9 ± 2.4	44.2 ± 2.4		42.0 ± 2.2	44.0 ± 2.4		43.4 ± 1.9	41.64 ± 3.7		42.9 ± 2.3	44.0 ± 2.7	
Tumour size (n)	31	54	NS (0.063)	29	53	NS (0.680)	71	14	NS (0.220)	51	33	NS (0.582)
Mean (cm)	2.20 ± 0.22	3.10 ± 0.27		2.59 ± 0.32	2.67 ± 0.24		2.86 ± 0.22	2.29 ± 0.43		2.87 ± 0.28	2.49 ± 0.23	
Gender (n)	33	56	NS (0.148)	31	55	NS (0.232)	75	14	NS (0.350)	54	34	NS (0.175)
Female (%)	26 (78.8)	50 (89.3)		28 (90.3)	45 (81.8)		63 (84.0)	13 (92.9)		44 (81.5)	31 (91.2)	
Male (%)	7 (21.2)	6 (10.7)		3 (9.7)	10 (18.2)		12 (16.0)	1 (7.1)		10 (18.5)	3 (8.8)	
Capsule (n)	30	50	NS (0.263)	26	51	NS (0.099)	66	14	**0.037**	49	30	NS (0.128)
Positive (%)	18 (60.0)	25 (50.0)		17 (65.4)	24 (47.1)		39 (59.1)	4 (28.6)		29 (59.2)	13 (43.3)	
Capsular invasion (n)	17	23	NS (0.496)	15	23	NS (0.332)	37	3	NS (0.704)	28	11	NS (0.191)
Positive (%)	12 (70.6)	15 (65.2)		11 (73.3)	14 (60.9)		25 (67.6)	2 (66.7)		17 (60.7)	9 (81.8)	
Vascular invasion (n)	30	51	NS (0.516)	28	51	NS (0.245)	67	14	NS (0.060)	50	30	NS (0.261)
Positive (%)	17 (56.7)	30 (58.8)		14 (50.0)	31 (60.8)		42 (62.7)	5 (35.7)		30 (60.0)	15 (50.0)	
Lymph node metastasis (n)	31	54	NS (0.398)	30	53	NS (0.073)	71	14	NS (0.397)	51	33	NS (0.074)
Positive (%)	7 (22.6)	15 (27.8)		5 (16.7)	18 (34.0)		21 (29.6)	3 (21.4)		10 (19.6)	12 (36.4)	
Extrathyroidal extension (n)	30	51	**0.003**	29	50	**0.020**	67	13	NS (0.328)	48	32	NS (0.332)
Positive (%)	4 (13.3)	23 (45.1)		5 (17.2)	21 (42.0)		23 (34.3)	3 (23.1)		17 (35.4)	9 (28.1)	
Distant metastasis (n)	31	54	NS (0.096)	30	53	NS (0.401)	71	14	NS (0.397)	51	33	NS (0.657)
Positive (%)	–	5 (9.3)		1 (3.33)	4 (7.55)		5 (7.04))	–		2 (3.92)	2 (6.06)	
Lymphocytic thyroiditis (n)	32	51	**0.003**	30	51	NS (0.416)	69	14	NS (0.563)	49	33	NS (0.354)
Positive (%)	7 (21.9)	28 (54.9)		12 (40.0)	23 (45.1)		31 (44.9)	6 (42.9)		19 (38.8)	15 (45.5)	
Oncocytic (n)	31	50	NS (0.290)	28	51	**0.018**	67	14	NS (0.329)	48	32	NS (0.473)
Positive (%)	5 (16.1)	12 (24.0)		2 (7.14)	15 (29.4)		13 (19.4)	4 (28.6)		9 (18.8)	7 (21.9)	
*PAX8-PPARG* rearrangements (n)	33	56	NS (0.371)	31	55	NS (0.360)	75	14	NS (0.843)	54	34	NS (0.386)
Positive (%)	1 (3.03)	–		1 (3.23)	–		1 (1.33)	–		–	1 (2.94)	
*RET/PTC* rearrangements (n)	33	56	NS (0.493)	31	55	NS (0.174)	75	14	NS (0.617)	54	34	NS (0.160)
Positive (%)	5 (15.1)	10 (17.9)		3 (9.68)	11 (20.0)		12 (16.0)	2 (14.3)		7 (13.0)	8 (23.5)	
*RET/PTC1* rearrangement (n)	33	56	NS (0.618)	31	55	NS (0.163)	75	14	NS (0.425)	54	34	NS (0.070)
Positive (%)	4 (12.1)	7 (12.5)		2 (6.45)	9 (16.4)		10 (13.3)	1 (7.14)		4 (7.41)	7 (20.6)	
*RET/PTC3* rearrangement (n)	33	56	NS (0.629)	31	55	^1^	75	14	^1^	54	34	NS (0.614)
Positive (%)	–	1 (1.79)		–	–		–	–		1 (1.85)	–	
*BRAF* mutation (n)	33	56	**<0.001**	31	55	**0.031**	75	14	**0.025**	54	34	NS (0.331)
Positive (%)	–	18 (32.1)		3 (9.68)	16 (29.1)		19 (25.3)	–		13 (24.1)	6 (17.6)	
*NRAS* mutation (n)	33	56	NS (0.213)	31	55	NS (0.300)	75	14	NS (0.090)	54	34	**0.001**
Positive (%)	7 (21.2)	7 (12.5)		6 (19.4)	7 (12.7)		13 (17.3)	–		14 (25.9)	–	
*TERT* promoter mutation (n)	33	56	NS (0.244)	31	55	NS (0.706)	75	14	NS (0.595)	54	34	NS (0.669)
Positive (%)	–	3 (5.36)		1 (3.23)	2 (3.64)		3 (4.00)	–		2 (3.70)	1 (2.94)	

n, number of cases with available data; 1, no statistics were computed due to constant numbers of one feature

Bold values indicate the result was statistically significant

### Clinical and molecular associations with gain of *LCN2* expression

**Fig. 4:**
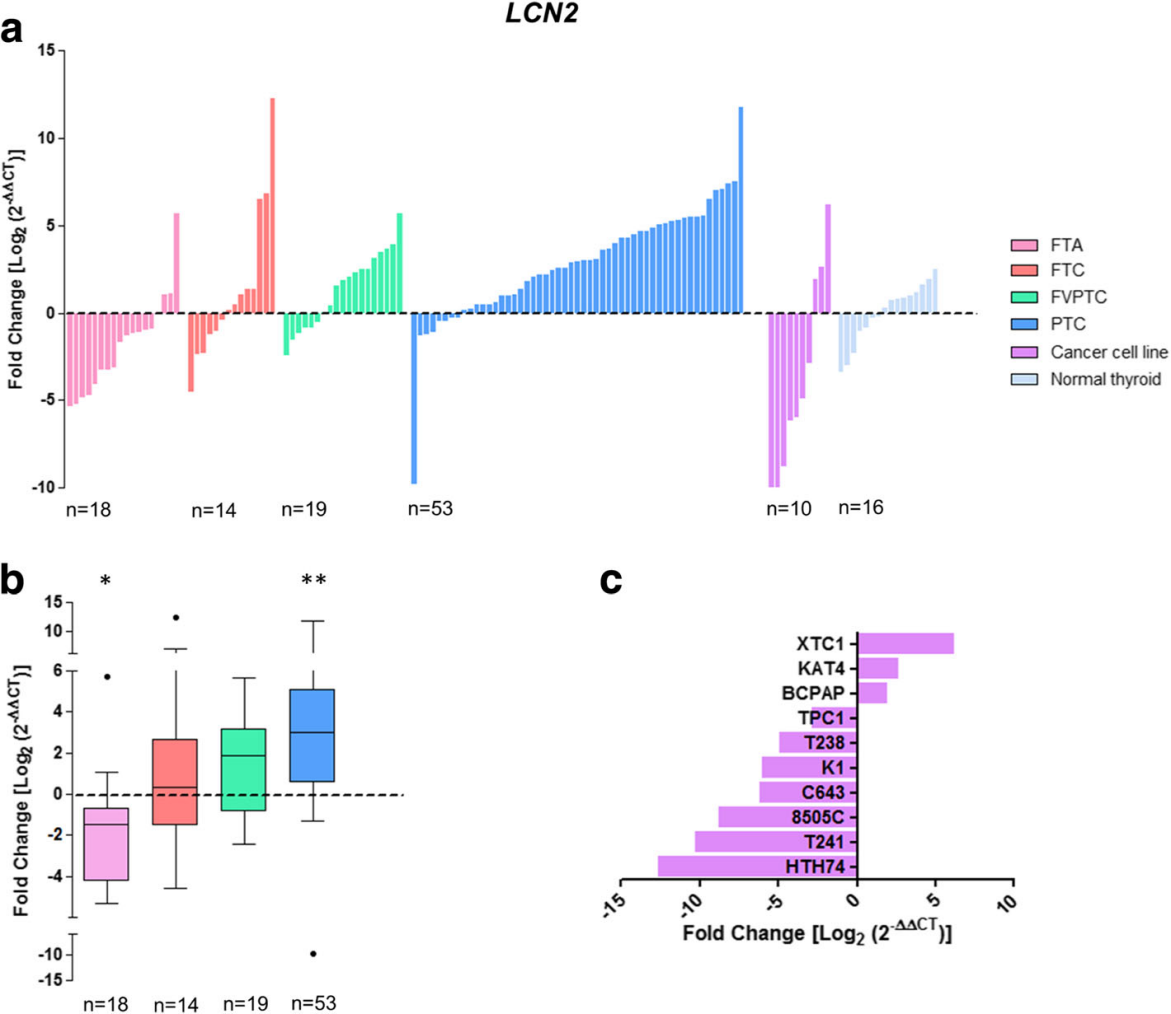
Differential gene expression of *LCN2* in thyroid tumours and normal tissues. Gene expression was measured in follicular thyroid adenoma (FTA), follicular thyroid cancer (FTC), follicular variant of papillary thyroid carcinoma (FVPTC), papillary thyroid carcinoma (PTC), normal thyroid tissues and thyroid cancer cell lines by real-time quantitative PCR. Histogram showing the gene expression of each sample ordered by expression levels (**a**). Box plot representation (median and Tukey whiskers) showing gene expression of each subgroup of thyroid tumours – FTA, FTC, FVPTC and PTC (**b**). Gene expression of the thyroid cancer cell lines (**c**). Gene expression was calibrated by the pool of normal thyroid tissues. Statistically significant values: *P = 0.013 **P < 0.001

*LCN2* expression levels were successful measured by qPCR in 86 DTC (14 FTC, 19 FVPTC and 53 PTC), 10 thyroid cancer cell lines, 18 FTA, and 16 matched thyroid normal tissues (Fig. 4a). Gain of *LCN2* expression was found in FTC, FVPTC and PTC (Fig. 4b), but the differences only achieved the threshold of statistical significance in PTC (*P* < 0.001). Three out of ten (30%) of the thyroid cancer cell lines showed gain of *LCN2* (Fig. 4c). At variance with DTC, significant loss of *LCN2* expression was found in FTA (*P* = 0.013; Fig. 4b). Gain of *LCN2* expression in DTC samples was significantly associated with the presence of extrathyroidal extension (*P* = 0.020), oncocytic features (*P* = 0.018), and the presence of *BRAF* mutations (*P* = 0.031; Table 3). Oncocytic pattern of the FTC was significantly associated with the gain of *LCN2* expression (*P* = 0.016; Additional file 12: Table S6). PTC with gain of *LCN2* expression were significantly larger (2.72 ± 0.30 cm) than PTC without gain of expression (1.76 ± 0.34 cm; *P* = 0.023; Additional file 14: Table S8). Although not statistically significant (*P* = 0.130), extrathyroidal extension was more frequent in PTC with gain (54%) than without gain (31%) of *LCN2* expression. No significant associations that could be related with gain of *LCN2* expression were observed in FVPTC.

### Clinical and molecular associations with loss of *CRABP1* expression

**Fig. 5:**
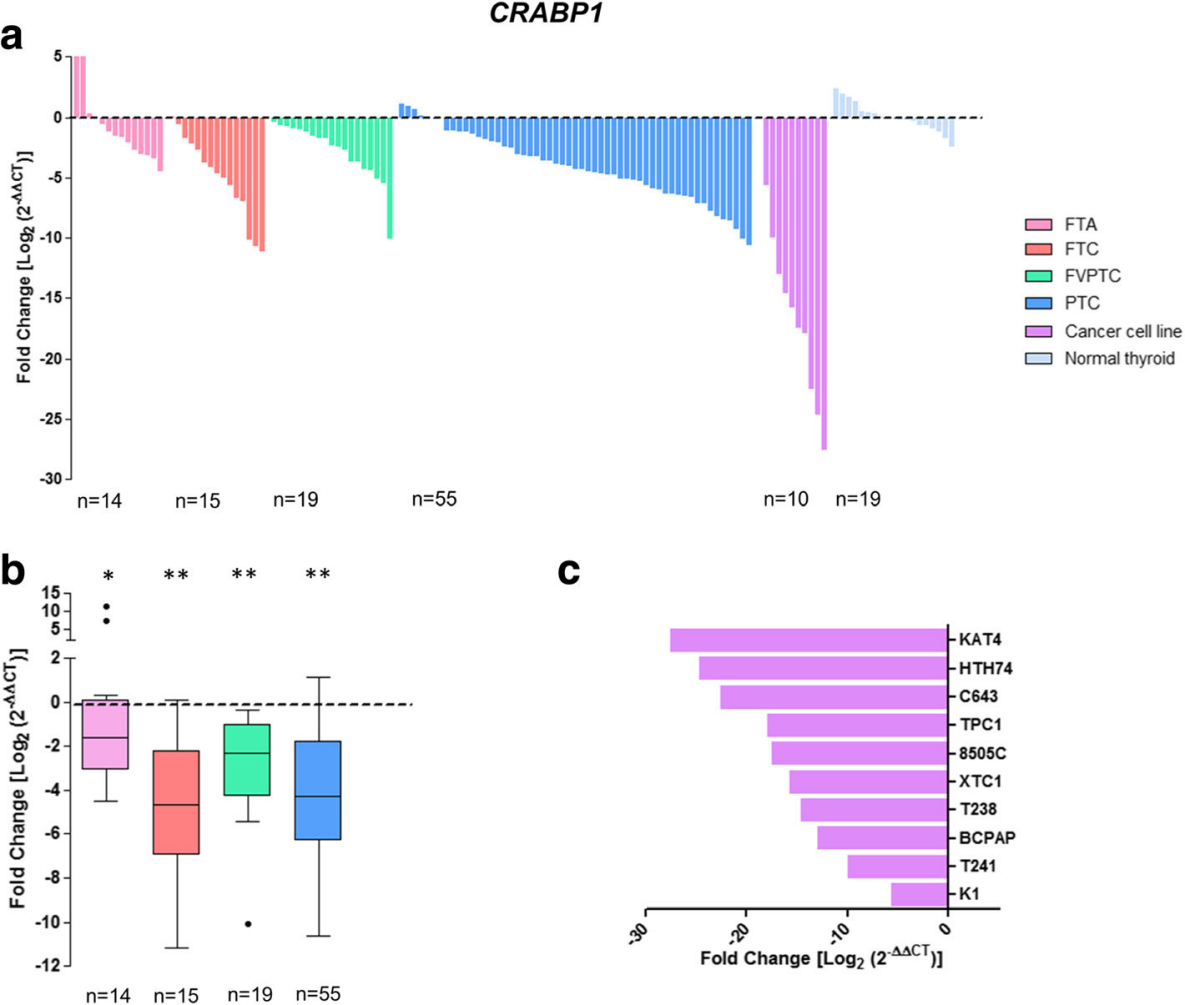
Differential gene expression of *CRABP1* in thyroid tumours and normal tissues. Gene expression was measured in follicular thyroid adenoma (FTA), follicular thyroid cancer (FTC), follicular variant of papillary thyroid carcinoma (FVPTC), papillary thyroid carcinoma (PTC), normal thyroid tissues and thyroid cancer cell lines by real-time quantitative PCR. Histogram showing the gene expression of each sample ordered by expression levels (**a**). Box plot representation (median and Tukey whiskers) showing gene expression of each subgroup of thyroid tumours – FTA, FTC, FVPTC and PTC (**b**). Gene expression of the thyroid cancer cell lines (**c**). Gene expression was calibrated by the pool of normal thyroid tissues. Statistically significant values: *P = 0.023 **P < 0.001

*CRABP1* expression levels were successful measured by qPCR in 89 DTC (15 FTC, 19 FVPTC and 55 PTC), 10 thyroid cancer cell lines, 14 FTA, and 19 matched thyroid normal tissues (Fig. 5a). Significant loss of *CRABP1* expression was detected in FTC, FVPTC and PTC (*P* < 0.001), and in FTA (*P* = 0.023; Fig. 5b). Notably, all the 10 thyroid cancer cell lines tested showed loss of *CRABP1* expression (Fig. 5c). Loss of *CRABP1* expression was associated with encapsulated DTC (*P* = 0.037). *BRAF* mutations (*P* = 0.025; Table 3) were only present in DTC with loss of *CRABP1* expression. No significant associations were found in FTC, FVPTC and PTC with loss of *CRABP1* expression.

### Clinical and molecular associations with loss of *CILP* expression

**Fig. 6:**
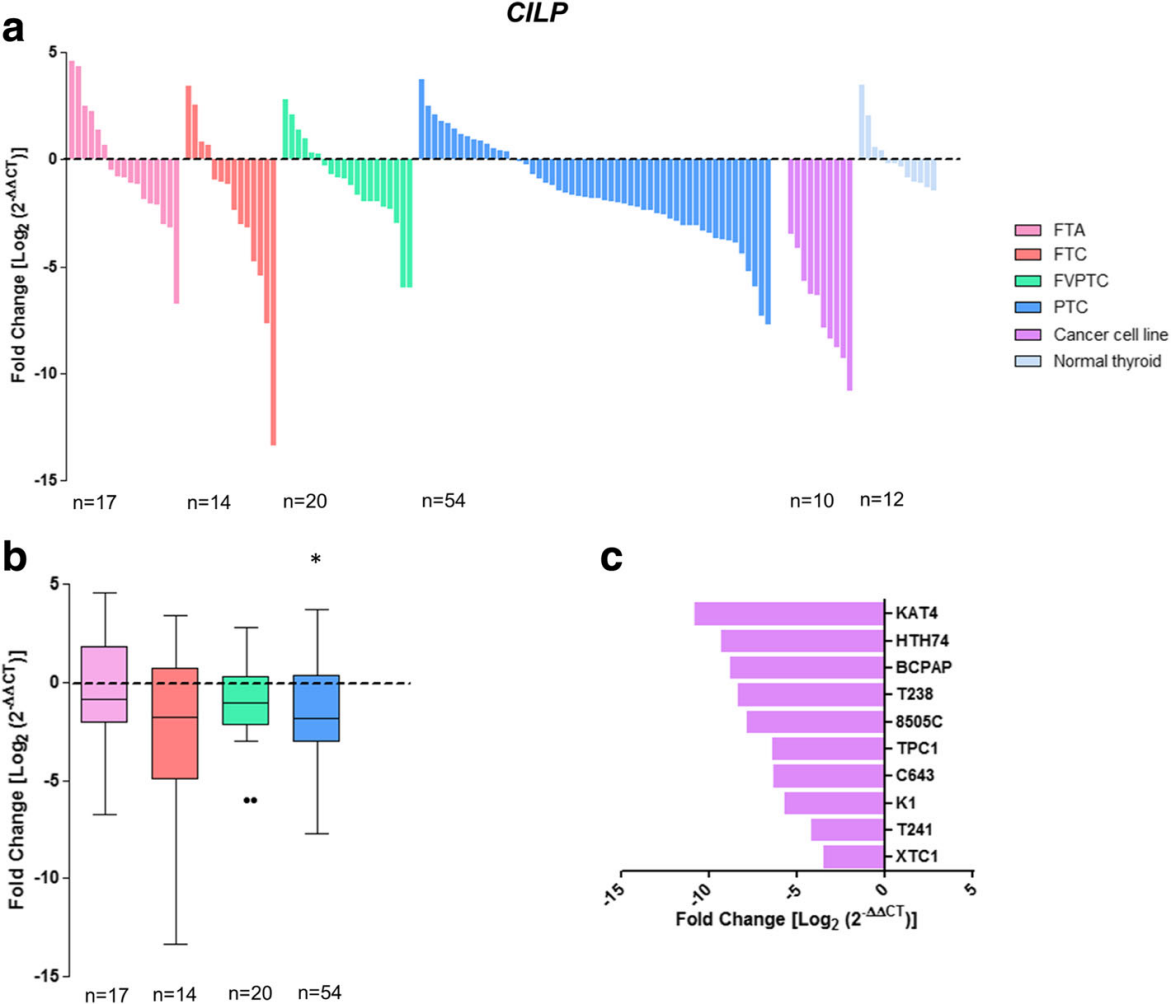
Differential gene expression of *CILP* in thyroid tumours and normal tissues. Gene expression was measured in follicular thyroid adenoma (FTA), follicular thyroid cancer (FTC), follicular variant of papillary thyroid carcinoma (FVPTC), papillary thyroid carcinoma (PTC), normal thyroid tissues and thyroid cancer cell lines by real-time quantitative PCR. Histogram showing the gene expression of each sample ordered by expression levels (**a**). Box plot representation (median and Tukey whiskers) showing gene expression of each subgroup of thyroid tumours – FTA, FTC, FVPTC and PTC (**b**). Gene expression of the thyroid cancer cell lines (**c**). Gene expression was calibrated by the pool of normal thyroid tissues. Statistically significant values: *P = 0.013

*CILP* expression levels were successfully measured by qPCR in 88 DTC (14 FTC, 20 FVPTC and 54 PTC), 10 thyroid cancer cell lines, 17 FTA, and twelve matched thyroid normal tissues (Fig. 6a). Loss of *CILP* was detected in FTC, FVPTC and PTC (Fig. 6b), but differences only attained the threshold of statistical significance in PTC (*P* = 0.013). All the ten thyroid cancer cell lines tested showed loss of *CILP* expression (Fig. 6c). *NRAS* mutations (*P* = 0.001; Table 3) were only present in DTC with loss of *CILP* expression. The same association was also found in FVPTC with loss of *CILP* expression (*P* = 0.043). No significant associations were found in FTC and PTC with loss of *CILP* expression.

### Association of thyroid cancer risk factors and gene expression

A regression model was performed with *C1QL1*, *LCN2*, *CRABP1* and *CILP* expression values for thyroid cancer prognostic factors: age of patients (> 45 years), tumour size (> 4 cm), and presence of extrathyroidal extension of the tumour (Table 4). Distant metastasis as prognostic factor was not considered for the computation in the regression models due to the reduced number of thyroid cancers with distant metastasis in the present series.

**Table 4:** Associations of fold change in gene expression with prognostic factors in differentiated thyroid cancer

		Age (≥ 45 years)		Tumour size (> 4 cm)		Extrathyroidal extension			
	Expression level (fold change)	Univariate analysis		Univariate analysis		Univariate analysis		Multivariate analysis	
		OR (95% CI)	*P* value	OR (95% CI)	*P* value	OR (95% CI)	*P* value	OR (95% CI)	*P* value
*C1QL1*	Gain (>1)	2.02 (0.835–4.88)	NS (0.119)	4.60 (0.963–21.9)	NS (0.056)	5.34 (1.63–17.5)	**0.006**	4.86 (1.43–16.5)	**0.011**
*LCN2*	Gain (>1)	1.17 (0.484–2.83)	NS (0.726)	0.730 (0.209–2.55)	NS (0.622)	3.48 (1.14–10.6)	**0.029**	3.39 (1.06–10.9)	**0.039**
*CRABP1*	Loss (<−1)	0.622 (0.197–1.97)	NS (0.419)	1.345 (0.268–6.75)	NS (0.719)	1.74 (0.436–6.96)	NS (0.432)	–	–
*CILP*	Loss (<−1)	0.481 (0.201–1.15)	NS (0.100)	2.75 (0.705–10.7)	NS (0.145)	1.40 (0.530–3.70)	NS (0.496)	–	–

Bold values indicate the result was statistically significant

In the univariate analysis, gain of the *C1QL1* [odds ratio (OR) = 5.34; *P* = 0.006] and *LCN2* (OR = 3.48; *P* = 0.029) expression were significantly associated with the extrathyroidal extension of the tumour. In the multivariate model, gain of *C1QL1* (OR = 4.86; *P* = 0.011) and *LCN2* (OR = 3.39; *P* = 0.039) expression were independent predictive factors for extrathyroidal extension in DTC. Additionally, gain of *C1QL1* expression was observed in the larger (> 4 cm) DTC (OR = 4.60), but the difference was only borderline in terms of statistical significance (*P* = 0.056). No associations were found between *CRABP1* and *CILP* expression and prognostic factors of thyroid cancer.

## Discussion

The clinical behaviour of thyroid cancer is still difficult to predict and clinical and histopathological prognostic factors remain the key elements for diagnosis and prognosis of the patients [4]. Due to the scarcity of molecular biomarkers that can predict the clinical behaviour of thyroid tumours, we used the high-throughput paired-end RNA-seq technology to progress in this subject. Until now, few RNA-seq studies on thyroid cancer have been published [9, 20–23], but none in FTC. In this pioneering study using FTC as a model, we gave a particular attention towards the identification of genes differentially expressed in mFTC and wFTC.

In total, 17 genes were found to be differentially expressed in mFTC and wFTC. After customized filtering and validation of expression values by qPCR, *C1QL1*, *LCN2*, *CRABP1* and *CILP* genes were selected for further validations in a larger series of DTC, using FTAs and a pool of normal thyroid tissues for comparison.

Remarkably, *CRABP1* was differentially expressed between DTC, FTA and normal thyroid tissue. Expression of *CRABP1* was significantly lower in FTC, FVPTC and PTC and in all ten thyroid cancer cell lines than in normal thyroid and FTA. In the ROC analysis, AUC for the *CRABP1* had the highest value (0.902), in the comparison of DTC versus normal thyroid. Gene expression from thyroid samples in TCGA reinforced *CRABP1* as biomarker in DTC.

*CRABP1* (cellular retinoic acid binding protein1) encodes a specific binding protein for a vitamin A family member and is thought to play an important role in retinoic acid-mediated differentiation and proliferation processes. Loss of *CRABP1* expression in thyroid cancer was shown in previous studies [24, 25], and hypermethylation of *CRABP1* promoter CpG islands has been shown as a possible explanation for its reduced expression in thyroid cancer [26] and in other human cancers [27–29]. Our results confirm the potential of *CRABP1* as a biomarker of DTC, based in a large number of DTC (*n* = 89) encompassing several subtypes – FTC, FVPTC and PTC, and controlled by a pool of normal thyroid tissues and of benign tumours – FTA. Our results suggest that *CRABP1* should be tested in order to see if it may be used in FNAB, for the differential diagnosis of the thyroid nodules displaying morphological features suspicious of malignancy. In contrast to results obtained in other human cancers [30, 31], we did not find any significant association between *CRABP1* expression and several well established prognostic factor in thyroid cancer.

*CILP* was found differentially expressed between DTC and normal thyroid tissue. Loss of *CILP* expression was observed in FTC, FVPTC and PTC, but the difference was only significant for the PTC. Notably, all thyroid cancer cell lines had loss of *CILP* expression. *NRAS* mutations were only present in DTC with loss of *CILP* expression. No associations were observed between *CILP* expression and other prognostic factors of thyroid cancer.

*CILP* (cartilage intermediate layer protein) can act as a negative regulator of TGFβ and inhibitory effect of *CILP* on TGFβ signalling increases with cartilage degeneration [32]. To the best of our knowledge there are no reports on record evidencing a possible relationship between *CILP* expression and cancer. Based on the data obtained in our study, it is not clear that *CILP* may be used as a good biomarker for DTC in spite of the aforementioned differentiated expression between PTC and normal thyroid tissue. The study of a larger series is required to clarify this issue.

Similar to the *CRABP1*, *LCN2* was found differentially expressed between DTC, FTA and normal thyroid tissue. Gain of *LCN2* expression was detected in FTC, FVPTC and PTC, but this gain only obtained the threshold of statistical significant in PTC. In the ROC analysis, AUC for the *LCN2* had the highest value (0.898), in the comparison of DTC versus FTA. This finding suggests that *LCN2* overexpression may be an interesting biomarker of DTC. Gain of *LCN2* expression in DTC was significantly associated with the presence of extrathyroidal extension, oncocytic features and presence of *BRAF* mutation. In addition, gain of *LCN2* expression in PTC was significantly associated with larger tumour size. Gene expression found in thyroid samples from TCGA confirmed *LCN2* as biomarker in DTC.

*LCN2* is a small secreted glycoprotein from the lipocalins family proteins that is important in the protection against bacterial infection and in the modulation of oxidative stress [33]. Consistent with our findings, *LCN2* protein overexpression has previously been found in a small series of thyroid cancers [34]. Our data show that higher *LCN2* expression (OR = 3.48; *P* = 0.029) is significantly associated with extrathyroidal extension, a prognostic factor that is strongly associate with progression of thyroid cancer. Functional studies in thyroid cancer cell lines also support this assumption; ectopic expression of *LCN2* in thyroid cancer cell line leads to an increase of its metastatic behaviour [35]. *LCN2* has been indicated as a potential diagnostic and prognostic cancer biomarker in other cancer models [reviewed in [33]]. Our results indicate that *LCN2* expression may also serve as a useful biomarker in DTC, namely for the FNAB diagnosis of thyroid nodules.

*C1QL1* was found differentially expressed between DTC and normal thyroid tissue. We observed a clear cut gain of *C1QL1* expression in FTC, FVPTC and PTC. The relative low number of FTC of the present series can justify the lack of statistical significance verified in the comparison of FTC with normal thyroid tissue regarding *C1QL1* expression. Gain of *C1QL1* expression was significantly associated with the presence of extrathyroidal extension in DTC. In PTC, tumours with gain of *C1QL1* expression were significantly larger than tumours without *C1QL1* overexpression. Since extrathyroidal extension and tumour size are major prognostic factors of thyroid cancer it may be advance that gain of expression of *C1QL1* will probably be a good indicator of clinical evolution. Notably, gain of *C1QL1* expression was significantly higher in wFTC than in mFTC, reinforcing the assumption that gain of *C1QL1* expression is related with thyroid cancer progression. Although we found an association of *C1QL1* overexpression and lymphocytic thyroiditis in DTC, further statistical analyses revealed no significant differences of *C1QL1* expression between the subtypes of DTC with lymphocytic thyroiditis and without lymphocytic thyroiditis (*P* = 0.316). Gene expression from thyroid samples in TCGA reinforced C*1QL1* as biomarker in DTC.

*C1QL1* is member of a subfamily of small secreted proteins of unknown function -C1q-like that are expressed almost exclusively in brain, and produced in differential patterns by specific types of neurons [36]. There are no current reports establishing a possible relation between *C1QL1* expression and cancer. Despite this, our study showed a significant association between higher *C1QL1* expression (OR = 5.34; *P* = 0.006) and extrathyroidal extension. Additionally, gain of *C1QL1* expression was observed in the larger (> 4 cm) thyroid cancers (OR = 4.60; *P* = 0.056), thus reinforcing the importance of *C1QL1* expression as predictor of thyroid cancer progression. Further studies in larger patient-series are necessary to confirm the statistical significance of our finding with regard to *C1QL1* expression.

Afirma™ Gene Expression Classifier (GEC) and ThyroSeq® v.2 are diagnostic tools commercially available and provide accurate classification of most thyroid nodules into benign or malignant in FNAB with undetermined diagnostic [37–41]. Both assays have high negative (NPV) and/or positive predictive values (PPV) in indicating malignancy of thyroid nodules [39]. These diagnostic tools avoid unnecessary surgeries reducing costs and risks for the patients. Although very useful, these technologies are based in a panel of numerous biomarkers, with high costs and not used broadly. Additionally, the use of molecular data for predict the prognostic of thyroid cancer patients remains controversial [42] and the identification of biomarkers with prognostic potential (as we describe here for *C1QL1* and *LCN2*) can further improve the design of this kind of assays. Curiously, the Afirma™ GEC and ThyroSeq® v.2 panels do not include any gene validated in our data, reinforcing the relevance and novelty of the biomarkers described here. A possible limitation of the present study was the relatively small discovery set used in the RNA-seq analysis, however, to obviate that, the results were validated by RT-PCR in a series of 118 thyroid tumours and 19 normal thyroid samples and by bioinformatics expression analysis in 393 PTC and 59 thyroid normal samples using the TCGA dataset [9].

## Conclusions

In conclusion, we found that expression of the individual genes *CRABP1* and *LCN2* may be useful biomarkers in DTC, able to improve FNAB differential diagnosis of benign versus malignant tumours. We also found that *LCN2* and *C1QL1* are independent predictors of extrathyroidal extension in DTC. The utility of these biomarkers should be evaluated in larger patient series and, in case our findings are confirmed, it will be very interesting to witness the development of tools for the diagnosis and/or prognostic of thyroid cancer using them.

## Additional files

Additional file 1: Clinicopathological features and genetic alterations of the differentiated thyroid carcinoma (DTC) series. (DOCX 12 kb)

Additional file 2: Table S1. Genes differentially expressed between minimally (mFTC) and widely invasive follicular thyroid carcinoma (wFTC). (DOCX 14 kb)

Additional file 3: Table S2. Transcripts differentially expressed between minimally (mFTC) and widely invasive follicular thyroid carcinoma (wFTC). (DOCX 14 kb)

Additional file 4: Figure S1. Ratio of cancer/normal tissue gene expression levels of the FTC cases used in RNA-seq. Gene expression was measured by real-time quantitative PCR in the FTC (cases 1–4) used in high-throughput paired-end RNA-seq. FTC, follicular thyroid carcinoma; mFTC, minimally invasive FTC; wFTC, widely invasive FTC. (JPEG 355 kb)

Additional file 5: Figure S2. Number of fusion genes identified in FTC cases using stringent requirements in RNA-seq data. The identified fused sequences were filtered in a customized manner for nomination of fusion genes for further experimental validation by reverse transcription-PCR and Sanger sequencing. Case 1 and 2 are widely invasive FTC, and case 3 and 4 are minimally invasive FTC. FTC, follicular thyroid carcinoma. (JPEG 351 kb)

Additional file 6: Table S3. Fusion genes selected by customized filtering steps and experimentally validated by RT-PCR and Sanger sequencing. FTC, follicular thyroid carcinoma; ORF, open reading frame. (DOCX 15 kb)

Additional file 7: Table S4. Oligonucleotide primers used in RT-PCR to detect the fusion genes. (DOCX 13 kb)

Additional file 8: Prediction and experimental validation of the fusion genes expressed in follicular thyroid carcinomas used in RNA-sequencing. (DOCX 17 kb)

Additional file 9: Figure S3. Differential gene expression of *C1QL1*, *LCN2*, *CRABP1* and *CILP* in thyroid tumours and normal tissues. Gene expression of *C1QL1* (a), *LCN2* (b), *CRABP1* (c) and *CILP* (d) genes was measured by real-time quantitative PCR in normal thyroid (NT) tissues, follicular thyroid adenoma (FTA) and differentiated thyroid cancer (DTC). Each dot represents the mean of gene expression of each sample. The lines represent the averages. Statistical significance values: *, *P* = 0.002; **, *P* = 0.005; ***, *P* = 0.013; ****, *P* < 0.001; *****, *P* = 0.022; ******, *P* = 0.018. (JPEG 417 kb)

Additional file 10: Table S5. Receiver Operating Characteristics (ROC) curve analysis. ROC curves for individual biomarkers were generated using log2 (2^-ΔΔCT^) gene expression values and thyroid tissue type [differentiated thyroid carcinoma (DTC) and normal or DTC and follicular thyroid adenoma (FTA)] as input. For evaluation of the combined biomarker panel the sum of log2 (2^-ΔΔCT^) expression values from genes with gain (*C1QL1* and *LCN2*) and loss (*CRABP1* and *CILP*) in DTC were used. AUC, area under the curve; CI, confidence interval. (DOCX 14 kb)

Additional file 11: Figure S4. Gene expression of *C1QL1*, *LCN2*, *CRABP1* and *CILP* in thyroid cancers available in TCGA. Gene expression values (normalized read counts) of *C1QL1* (a), *LCN2* (b), *CRABP1* (c) and *CILP* (d) in differentiated thyroid cancer (DTC) and normal thyroid (NT) tissues available in The Cancer Genome Atlas (TCGA). Each dot represents the gene expression value of each sample. The lines represent the averages. Statistical significance values: *, *P* < 0.001. (JPEG 518 kb)

Additional file 12: Table S6. Clinicopathological and genetic data of the FTC classified by classes based on gene expression. (DOCX 18 kb)

Additional file 13: Table S7. Clinicopathological and genetic data of the FVPTC classified by classes based on gene expression. (DOCX 17 kb)

Additional file 14: Table S8. Clinicopathological and genetic data of the PTC classified by classes based on gene expression. (DOCX 17 kb)

## Abbreviations

CT: Cycle threshold
DTC: Differentiated thyroid carcinoma
FISH: Fluorescence in situ hybridization
FNAB: Fine needle aspiration biopsy
FTA: Follicular thyroid adenoma
FTC: Follicular thyroid carcinoma
HPRT: Hypoxanthine phosphoribosyltransferase
mFTC: Minimally invasive FTC
PTC: Papillary thyroid carcinoma
RNA-seq: RNA sequencing
ROC: Receiver Operating Characteristics
RT-PCR: Reverse-transcription PCR
TCGA: The Cancer Genome Atlas;
wFTC: Widely invasive FTC

## References

[1] Howlader N, Noone AM, Krapcho M, Neyman N, Aminou R, Waldron W, Altekruse SF, Kosary CL, Ruhl J, Tatalovich Z, Cho H, Mariotto A, Eisner MP, Lewis DR, Chen HS, Feuer EJ, Cronin KA (eds). SEER Cancer Statistics Review, 1975-2009 (Vintage 2009 Populations), National Cancer Institute. Bethesda, MD, http://seer.cancer.gov/csr/1975_2009_pops09/, based on November 2011 SEER data submission, posted to the SEER web site, April 2012.

[2] Kondo T, Ezzat S, Asa SL (2006). Pathogenetic mechanisms in thyroid follicular-cell neoplasia. Nat Rev Cancer.

[3] DeLellis RA, Lloyd RV, Heitz PU, Eng C. WHO classification of Tumours. Pathology and Genetics of Tumours of Endocrine Organs. Lyon: IARC Press; 2004.

[4] Soares P, Celestino R, Melo M, Fonseca E, Sobrinho-Simoes M (2014). Prognostic biomarkers in thyroid cancer. Virchows Arch.

[5] Melo M, da Rocha AG, Vinagre J, Batista R, Peixoto J, Tavares C, Celestino R, Almeida A, Salgado C, Eloy C, et al. TERT promoter mutations are a major indicator of poor outcome in differentiated thyroid carcinomas. J Clin Endocrinol Metab. 2014;99:E754–765. 10.1210/jc.2013-3734 PMC4191548 24476079

[6] Qureshi AA, Collins VP, Jani P (2013). Genomic differences in benign and malignant follicular thyroid tumours using 1-Mb array-comparative genomic hybridisation. Eur Arch Otorhinolaryngol.

[7] Sobrinho-Simoes M, Eloy C, Magalhaes J, Lobo C, Amaro T (2011). Follicular thyroid carcinoma. Mod Pathol.

[8] Collini P, Sampietro G, Rosai J, Pilotti S (2003). Minimally invasive (encapsulated) follicular carcinoma of the thyroid gland is the low-risk counterpart of widely invasive follicular carcinoma but not of insular carcinoma. Virchows Arch.

[9] Cancer Genome Atlas Research (2014). N. Integrated genomic characterization of papillary thyroid carcinoma. Cell.

[10] Trapnell C, Williams BA, Pertea G, Mortazavi A, Kwan G, van Baren MJ, Salzberg SL, Wold BJ, Pachter L (2010). Transcript assembly and quantification by RNA-Seq reveals unannotated transcripts and isoform switching during cell differentiation. Nat Biotechnol.

[11] Trapnell C, Pachter L, Salzberg SL (2009). TopHat: discovering splice junctions with RNA-Seq. Bioinformatics.

[12] Celestino R, Sigstad E, Lovf M, Thomassen GO, Groholt KK, Jorgensen LH, Berner A, Castro P, Lothe RA, Bjoro T (2012). Survey of 548 oncogenic fusion transcripts in thyroid tumors supports the importance of the already established thyroid fusions genes. Genes Chromosomes Cancer.

[13] Vinagre J, Almeida A, Populo H, Batista R, Lyra J, Pinto V, Coelho R, Celestino R, Prazeres H, Lima L (2013). Frequency of TERT promoter mutations in human cancers. Nat Commun.

[14] Livak KJ, Schmittgen TD (2001). Analysis of relative gene expression data using real-time quantitative PCR and the 2(−Delta Delta C(T)) method. Methods.

[15] Cancer Genome Atlas Research Network (2014). Integrated genomic characterization of papillary thyroid carcinoma. Cell.

[16] Li B, Dewey CNRSEM (2011). Accurate transcript quantification from RNA-Seq data with or without a reference genome. BMC Bioinformatics.

[17] Giordano TJ, Au AY, Kuick R, Thomas DG, Rhodes DR, Wilhelm KG, Vinco M, Misek DE, Sanders D, Zhu Z (2006). Delineation, functional validation, and bioinformatic evaluation of gene expression in thyroid follicular carcinomas with the PAX8-PPARG translocation. Clin Cancer Res.

[18] He H, Jazdzewski K, Li W, Liyanarachchi S, Nagy R, Volinia S, Calin GA, Liu CG, Franssila K, Suster S (2005). The role of microRNA genes in papillary thyroid carcinoma. Proc Natl Acad Sci U S A.

[19] Vasko V, Espinosa AV, Scouten W, He H, Auer H, Liyanarachchi S, Larin A, Savchenko V, Francis GL, de la Chapelle A (2007). Gene expression and functional evidence of epithelial-to-mesenchymal transition in papillary thyroid carcinoma invasion. Proc Natl Acad Sci U S A.

[20] Leeman-Neill RJ, Kelly LM, Liu P, Brenner AV, Little MP, Bogdanova TI, Evdokimova VN, Hatch M, Zurnadzy LY, Nikiforova MN (2014). ETV6-NTRK3 is a common chromosomal rearrangement in radiation-associated thyroid cancer. Cancer.

[21] Ricarte-Filho JC, Li S, Garcia-Rendueles ME, Montero-Conde C, Voza F, Knauf JA, Heguy A, Viale A, Bogdanova T, Thomas GA (2013). Identification of kinase fusion oncogenes in post-Chernobyl radiation-induced thyroid cancers. J Clin Invest.

[22] Zhang Y, Yu J, Lee C, Xu B, Sartor MA, Koenig RJ (2015). Genomic binding and regulation of gene expression by the thyroid carcinoma-associated PAX8-PPARG fusion protein. Oncotarget.

[23] Smallridge RC, Chindris AM, Asmann YW, Casler JD, Serie DJ, Reddi HV, Cradic KW, Rivera M, Grebe SK, Necela BM (2014). RNA sequencing identifies multiple fusion transcripts, differentially expressed genes, and reduced expression of immune function genes in BRAF (V600E) mutant vs BRAF wild-type papillary thyroid carcinoma. J Clin Endocrinol Metab.

[24] Hawthorn L, Stein L, Varma R, Wiseman S, Loree T, Tan D (2004). TIMP1 and SERPIN-A overexpression and TFF3 and CRABP1 underexpression as biomarkers for papillary thyroid carcinoma. Head Neck.

[25] Fontaine JF, Mirebeau-Prunier D, Raharijaona M, Franc B, Triau S, Rodien P, Goeau-Brissonniere O, Karayan-Tapon L, Mello M, Houlgatte R (2009). Increasing the number of thyroid lesions classes in microarray analysis improves the relevance of diagnostic markers. PLoS One.

[26] Huang Y, de la Chapelle A, Pellegata NS (2003). Hypermethylation, but not LOH, is associated with the low expression of MT1G and CRABP1 in papillary thyroid carcinoma. Int J Cancer.

[27] Berg M, Hagland HR, Soreide K (2014). Comparison of CpG island methylator phenotype (CIMP) frequency in colon cancer using different probe- and gene-specific scoring alternatives on recommended multi-gene panels. PLoS One.

[28] Wang F, Yang Y, Fu Z, Xu N, Chen F, Yin H, Lu X, Shen R, Differential LC. DNA methylation status between breast carcinomatous and normal tissues. Biomed Pharmacother. 2014; 10.1016/j.biopha.2014.07.014 25070394

[29] Tanaka K, Imoto I, Inoue J, Kozaki K, Tsuda H, Shimada Y, Aiko S, Yoshizumi Y, Iwai T, Kawano T, Inazawa J (2007). Frequent methylation-associated silencing of a candidate tumor-suppressor, CRABP1, in esophageal squamous-cell carcinoma. Oncogene.

[30] Kainov Y, Favorskaya I, Delektorskaya V, Chemeris G, Komelkov A, Zhuravskaya A, Trukhanova L, Zueva E, Tavitian B, Dyakova N (2014). CRABP1 provides high malignancy of transformed mesenchymal cells and contributes to the pathogenesis of mesenchymal and neuroendocrine tumors. Cell Cycle.

[31] Miyake T, Ueda Y, Matsuzaki S, Miyatake T, Yoshino K, Fujita M, Nomura T, Enomoto T, Kimura T (2011). CRABP1-reduced expression is associated with poorer prognosis in serous and clear cell ovarian adenocarcinoma. J Cancer Res Clin Oncol.

[32] Seki S, Kawaguchi Y, Chiba K, Mikami Y, Kizawa H, Oya T, Mio F, Mori M, Miyamoto Y, Masuda I (2005). A functional SNP in CILP, encoding cartilage intermediate layer protein, is associated with susceptibility to lumbar disc disease. Nat Genet.

[33] Chakraborty S, Kaur S, Guha S, Batra SK (1826). The multifaceted roles of neutrophil gelatinase associated lipocalin (NGAL) in inflammation and cancer. Biochim Biophys Acta.

[34] Iannetti A, Pacifico F, Acquaviva R, Lavorgna A, Crescenzi E, Vascotto C, Tell G, Salzano AM, Scaloni A, Vuttariello E (2008). The neutrophil gelatinase-associated lipocalin (NGAL), a NF-kappaB-regulated gene, is a survival factor for thyroid neoplastic cells. Proc Natl Acad Sci U S A.

[35] Volpe V, Raia Z, Sanguigno L, Somma D, Mastrovito P, Moscato F, Mellone S, Leonardi A, Pacifico FNGAL (2013). Controls the metastatic potential of anaplastic thyroid carcinoma cells. J Clin Endocrinol Metab.

[36] Iijima T, Miura E, Watanabe M, Yuzaki M (2010). Distinct expression of C1q-like family mRNAs in mouse brain and biochemical characterization of their encoded proteins. Eur J Neurosci.

[37] Alexander EK, Kennedy GC, Baloch ZW, Cibas ES, Chudova D, Diggans J, Friedman L, Kloos RT, LiVolsi VA, Mandel SJ (2012). Preoperative diagnosis of benign thyroid nodules with indeterminate cytology. N Engl J Med.

[38] Alexander EK, Schorr M, Klopper J, Kim C, Sipos J, Nabhan F, Parker C, Steward DL, Mandel SJ, Haugen BR (2014). Multicenter clinical experience with the Afirma gene expression classifier. J Clin Endocrinol Metab.

[39] Nikiforov YE, Carty SE, Chiosea SI, Coyne C, Duvvuri U, Ferris RL, Gooding WE, LeBeau SO, Ohori NP, Seethala RR (2015). Impact of the multi-gene ThyroSeq next-generation sequencing assay on cancer diagnosis in thyroid nodules with atypia of undetermined significance/follicular lesion of undetermined significance cytology. Thyroid.

[40] Nikiforov YE, Carty SE, Chiosea SI, Coyne C, Duvvuri U, Ferris RL, Gooding WE, Hodak SP, LeBeau SO, Ohori NP (2014). Highly accurate diagnosis of cancer in thyroid nodules with follicular neoplasm/suspicious for a follicular neoplasm cytology by ThyroSeq v2 next-generation sequencing assay. Cancer.

[41] McIver B, Castro MR, Morris JC, Bernet V, Smallridge R, Henry M, Kosok L, Reddi H (2014). An independent study of a gene expression classifier (Afirma) in the evaluation of cytologically indeterminate thyroid nodules. J Clin Endocrinol Metab.

[42] Kargi AY, Bustamante MP, Gulec S (2017). Genomic profiling of thyroid nodules: current role for ThyroSeq next-generation sequencing on clinical decision-making. Mol Imaging Radionucl Ther.

