# Supplementary material for: CRABP1, C1QL1 and LCN2 are biomarkers of differentiated thyroid carcinoma, and predict extrathyroidal extension
Source: BMC Cancer. 2018 Jan 10;18:68. doi: 10.1186/s12885-017-3948-3 (PMC5763897; doi:10.1186/s12885-017-3948-3)
Supplement: Supplementary file 1 — Clinicopathological features and genetic alterations of the differentiated thyroid carcinoma (DTC) series. (DOCX 12 kb) [file 12885_2017_3948_MOESM1_ESM.docx]

**Clinicopathological features and genetic alterations of the differentiated thyroid carcinoma (DTC) series**

In the series of 98 differentiated thyroid carcinomas (DTC; Table 1), the mean age of the patients was 43 ± 1.6 years, and the mean size of the tumours was 2.8 ± 0.18 cm. Patients with FTC (53 ± 3.9 years) were significantly older than patients with PTC (40 ± 2.1 years; *P* = 0.021). FTC (3.9 ± 0.46 cm) were significantly larger than FVPTC (2.4 ±0.30 cm; *P* = 0.022) and PTC (2.7 ± 0.24 cm; *P* = 0.034). The female/male gender ratio of the patients was 6:1. Forty-five DTC (45/88, 51%) had capsule, whereas capsular invasion occurred in 27 DTC (27/42, 64%). Vascular invasion occurred in 48 DTC (48/89, 54%), whereas FVPTC (5/21, 24%) had significantly less vascular invasion than FTC (11/15, 73%; *P* = 0.004) and PTC (32/53, 60%; *P* = 0.004). Lymph node metastases were present in 24 patients (24/93, 26%), presenting FVPTC (4/21, 19%) and PTC (20/57, 35%). Extrathyroidal extension was present in 27 DTC (27/88, 31%), occurring significantly more often in PTC (24/52, 46%) than in FTC (1/15, 7%; *P* = 0.004) and FVPTC (2/21, 10%; *P* = 0.002). Distant metastases were detected in five patients (5/93, 5%), one with a FTC (1/15, 7%), and four patients with PTC (4/57, 7%). Lymphocytic thyroiditis was detected in 40 patients (40/91, 44%), occurring in FCT (4/15, 27%), FVPTC (8/22, 37%) and PTC (28/54, 52%). Oncocytic pattern was present in 17 DTC (17/89, 19%): six FTC (6/15, 40%) and eleven PTC (11/53, 21%).

*PAX8-PPARG* rearrangements were detected in FTC (1/15, 7%) and FVPTC (1/23, 4%), and *RET/PTC* rearrangements were detected in FTC (1/15, 7%), FVPTC (3/23, 13%) and PTC (16/60, 22%). *BRAF* mutations were significantly more common in PTC (19/60, 32%) than in FVPTC (2/23, 9%; *P* = 0.025), and *NRAS* mutations were significantly more frequent in FTC (5/15; 33%) than in PTC (6/60; 10%; *P* = 0.037).

Patients with DTC positive for the *RET/PTC* rearrangement were significantly younger (35 ± 4.0 years) than patients with DTC negative for the *RET/PTC* (44 ± 1.7 years; *P* = 0.020). Despite the reduced number of *TERT* promoter mutations (n = 3) in this series, patients with DTC positive for the mutations were significantly older (72 ± 5.5 years) than patients with tumour without *TERT* promoter mutations (42 ± 1.6 years; *P* = 0.001). Additionally, tumours with *TERT* promoter mutations are larger (4.9 ± 1.6 cm) than the tumours without mutations (2.7 ± 0.18 cm; *P* = 0.032).
