# Supplementary material for: CRABP1, C1QL1 and LCN2 are biomarkers of differentiated thyroid carcinoma, and predict extrathyroidal extension
Source: BMC Cancer. 2018 Jan 10;18:68. doi: 10.1186/s12885-017-3948-3 (PMC5763897; doi:10.1186/s12885-017-3948-3)
Supplement: Supplementary file 2 — Genes differentially expressed between minimally (mFTC) and widely invasive follicular thyroid carcinoma (wFTC). (DOCX 14 kb) [file 12885_2017_3948_MOESM2_ESM.docx]

**Supplementary table 1** Genes differentially expressed between minimally (mFTC) and widely invasive follicular thyroid carcinoma (wFTC).

|  | Gene ID | Gene | Chr. | mFTC | wFTC | Fold change  (log2) | Q value | Gene ontology  (molecular function) |
| --- | --- | --- | --- | --- | --- | --- | --- | --- |
|  | ENSG00000167748 | *KLK1* | 19 | 0.083614 | 180.865 | 11.0789 | 0.00000199 | Serine-type endopeptidase activity |
|  | ENSG00000104725 | *NEFL* | 8 | 0.007367 | 12.2807 | 10.7031 | 0.000172 | Protein binding and structural constituent of cytoskeleton |
|  | ENSG00000174145 | *KIAA1239* | 4 | 0.011999 | 2.64975 | 7.78682 | 0.000283 | Molecular function unknown |
|  | ENSG00000182379 | *NXPH4* | 12 | 0.05929 | 10.1244 | 7.41584 | 0.001906 | Molecular function unknown |
|  | ENSG00000131094 | *C1QL1* | 17 | 0.051369 | 4.70064 | 6.51582 | 0.046856 | Molecular function unknown |
|  | ENSG00000142677 | *IL22RA1* | 1 | 11.5338 | 0.313374 | -5.20184 | 0.023093 | Interferon receptor activity  Interleukin-20 binding |
|  | ENSG00000165072 | *MAMDC2* | 9 | 27.6276 | 0.664279 | -5.37818 | 0.039133 | Glycosaminoglycan binding |
|  | ENSG00000138615 | *CILP* | 15 | 3.54412 | 0.033979 | -6.70465 | 0.002817 | Alkaline phosphatase activity  Nucleotide diphosphatase activity |
|  | ENSG00000152207 | *CYSLTR2* | 13 | 12.9294 | 0.11492 | -6.81389 | 0.000524 | Cysteinyl leukotriene receptor activity |
|  | ENSG00000141431 | *ASXL3* | 18 | 0.971358 | 0.008418 | -6.85037 | 0.024139 | DNA binding  Metal ion binding |
|  | ENSG00000166426 | *CRABP1* | 15 | 67.5458 | 0.225117 | -8.22905 | 0.001888 | Retinoic acid binding  Transporter activity |
|  | ENSG00000214145 | *LINC00887* | 3 | 20.558 | 0.063026 | -8.34954 | 0.039133 | Molecular function unknown |
