# Supplementary material for: CRABP1, C1QL1 and LCN2 are biomarkers of differentiated thyroid carcinoma, and predict extrathyroidal extension
Source: BMC Cancer. 2018 Jan 10;18:68. doi: 10.1186/s12885-017-3948-3 (PMC5763897; doi:10.1186/s12885-017-3948-3)
Supplement: Supplementary file 3 — Transcripts differentially expressed between minimally (mFTC) and widely invasive follicular thyroid carcinoma (wFTC). (DOCX 14 kb) [file 12885_2017_3948_MOESM3_ESM.docx]

**Supplementary table 2** Transcripts differentially expressed between minimally (mFTC) and widely invasive follicular thyroid carcinoma (wFTC).

|  | Transcript ID | Gene | Chr. | mFTC | wFTC | Fold change  (log2) | q value | Gene ontology  (molecular function) |
| --- | --- | --- | --- | --- | --- | --- | --- | --- |
|  | ENST00000301420 | *KLK1* | 19 | 0.083614 | 180.753 | 11.078 | 0.00000461 | Serine-type endopeptidase activity |
|  | ENST00000221169 | *NEFL* | 8 | 0.007367 | 12.2807 | 10.7031 | 0.000375 | Protein binding and structural constituent of cytoskeleton |
|  | ENST00000309447 | *KIAA1239* | 4 | 0.011999 | 2.64975 | 7.78682 | 0.000695 | Molecular function unknown |
|  | ENST00000450985 | *SLC6A17* | 1 | 0.016688 | 3.42221 | 7.67995 | 0.004338 | Neurotransmitter:sodium symporter activity |
|  | ENST00000349394 | *NXPH4* | 12 | 0.05929 | 10.1244 | 7.41584 | 0.004853 | Molecular function unknown |
|  | ENST00000277480 | *LCN2* | 9 | 0.316456 | 48.3541 | 7.25549 | 0.01633 | Small molecule binding  Transporter activity |
|  | ENST00000217233 | *TRIB3* | 20 | 1.11631 | 78.9564 | 6.14425 | 0.009131 | ATP binding, protein kinase activity and binding, transcription corepressor activity, ubiquitin protein ligase binding and regulator activity |
|  | ENST00000448044 | *ST6GAL1* | 3 | 167.299 | 8.19519 | -4.35151 | 0.011511 | Beta-galactoside alpha-2,6-sialyltransferase activity |
|  | ENST00000274938 | *SCUBE3* | 6 | 38.2495 | 1.18184 | -5.01633 | 0.004338 | Calcium ion binding  Protein binding |
|  | ENST00000270800 | *IL22RA1* | 1 | 11.5338 | 0.313374 | -5.20184 | 0.048597 | Interferon receptor activity  Interleukin-20 binding |
|  | ENST00000261883 | *CILP* | 15 | 3.54412 | 0.033979 | -6.70465 | 0.007378 | Alkaline phosphatase activity  Nucleotide diphosphatase activity |
|  | ENST00000282018 | *CYSLTR2* | 13 | 12.9294 | 0.11492 | -6.81389 | 0.001716 | Cysteinyl leukotriene receptor activity |
|  | ENST00000299529 | *CRABP1* | 15 | 67.3719 | 0.224968 | -8.22628 | 0.0000705 | Retinoic acid binding  Transporter activity |
