# Supplementary figures and images for: CRABP1, C1QL1 and LCN2 are biomarkers of differentiated thyroid carcinoma, and predict extrathyroidal extension
Source: BMC Cancer. 2018 Jan 10;18:68. doi: 10.1186/s12885-017-3948-3 (PMC5763897; doi:10.1186/s12885-017-3948-3)

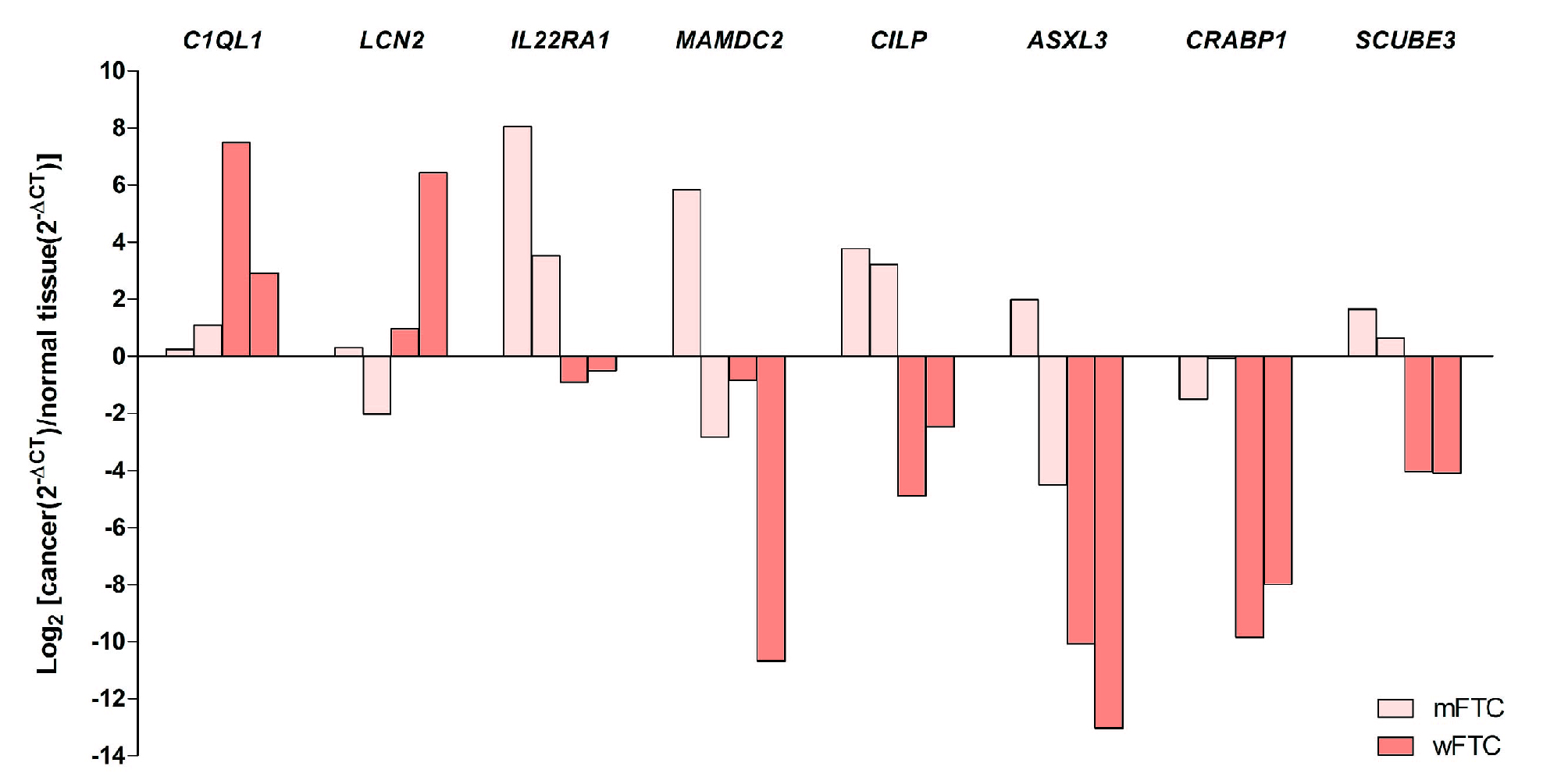

Supplement: Supplementary file 4 — Ratio of cancer/normal tissue gene expression levels of the FTC cases used in RNA-seq. Gene expression was measured by real-time quantitative PCR in the FTC (cases 1–4) used in high-throughput paired-end RNA-seq. FTC, follicular thyroid carcinoma; mFTC, minimally invasive FTC; wFTC, widely invasive FTC. (JPEG 355 kb) [file 12885_2017_3948_MOESM4_ESM.jpg]

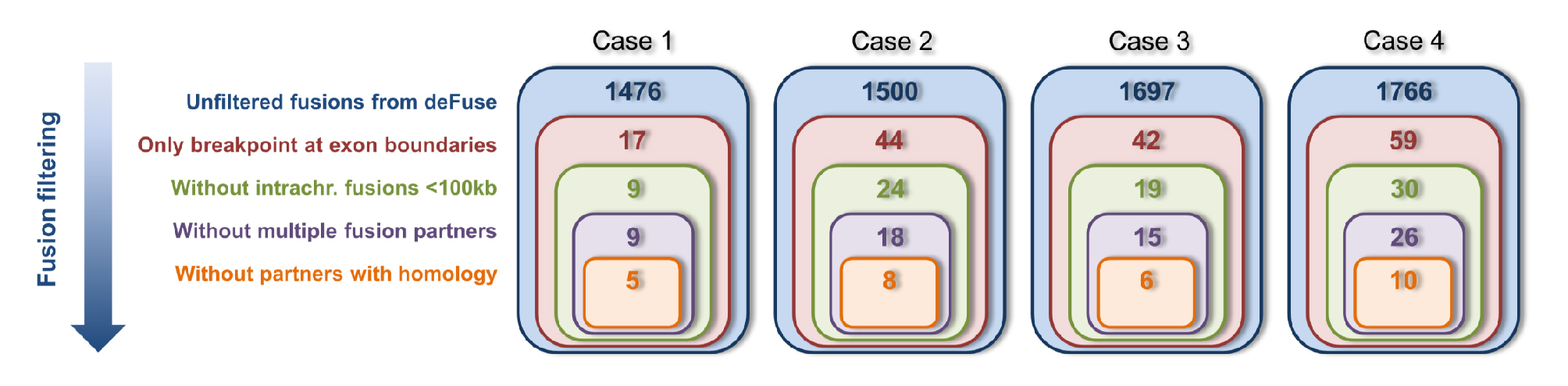

Supplement: Supplementary file 5 — Number of fusion genes identified in FTC cases using stringent requirements in RNA-seq data. The identified fused sequences were filtered in a customized manner for nomination of fusion genes for further experimental validation by reverse transcription-PCR and Sanger sequencing. Case 1 and 2 are widely invasive FTC, and case 3 and 4 are minimally invasive FTC. FTC, follicular thyroid carcinoma. (JPEG 351 kb) [file 12885_2017_3948_MOESM5_ESM.jpg]

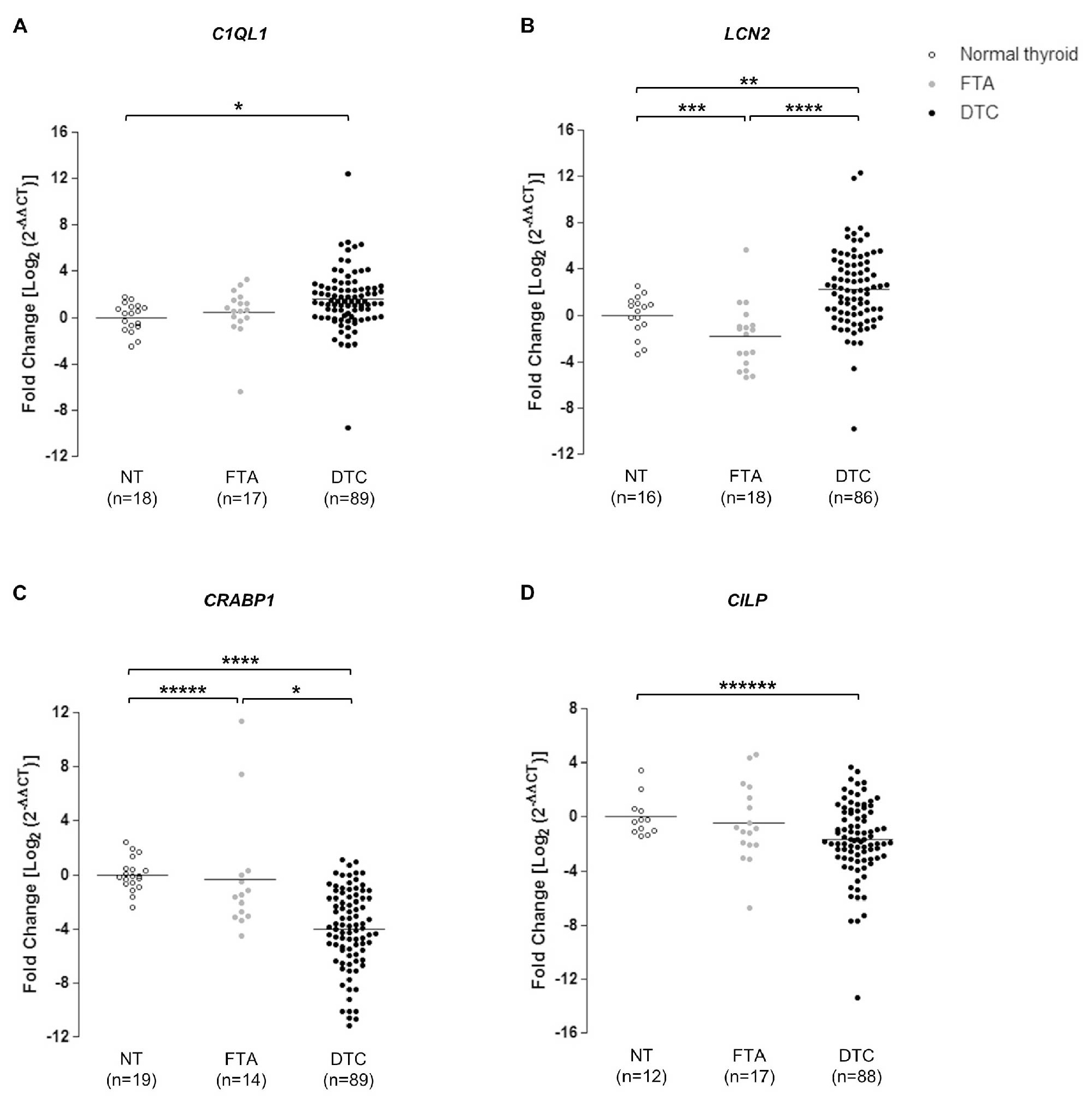

Supplement: Supplementary file 9 — Differential gene expression of C1QL1, LCN2, CRABP1 and CILP in thyroid tumours and normal tissues. Gene expression of C1QL1 (a), LCN2 (b), CRABP1 (c) and CILP (d) genes was measured by real-time quantitative PCR in normal thyroid (NT) tissues, follicular thyroid adenoma (FTA) and differentiated thyroid cancer (DTC). Each dot represents the mean of gene expression of each sample. The lines represent the averages. Statistical significance values: *, P = 0.002; **, P = 0.005; ***, P = 0.013; ****, P < 0.001; *****, P = 0.022; ******, P = 0.018. (JPEG 417 kb) [file 12885_2017_3948_MOESM9_ESM.jpg]

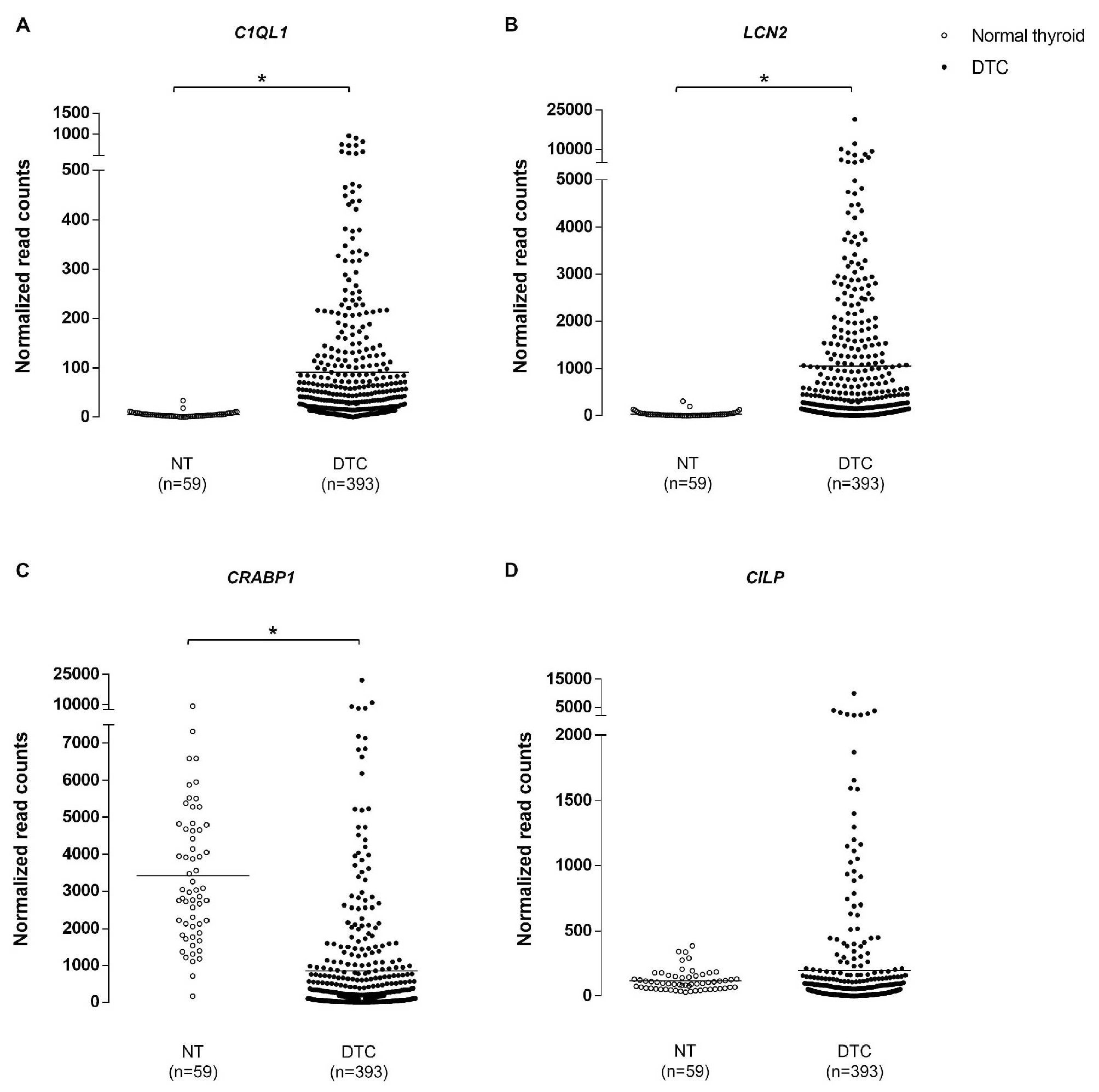

Supplement: Supplementary file 11 — Gene expression of C1QL1, LCN2, CRABP1 and CILP in thyroid cancers available in TCGA. Gene expression values (normalized read counts) of C1QL1 (a), LCN2 (b), CRABP1 (c) and CILP (d) in differentiated thyroid cancer (DTC) and normal thyroid (NT) tissues available in The Cancer Genome Atlas (TCGA). Each dot represents the gene expression value of each sample. The lines represent the averages. Statistical significance values: *, P < 0.001. (JPEG 518 kb) [file 12885_2017_3948_MOESM11_ESM.jpg]
