# Supplementary material for: CRABP1, C1QL1 and LCN2 are biomarkers of differentiated thyroid carcinoma, and predict extrathyroidal extension
Source: BMC Cancer. 2018 Jan 10;18:68. doi: 10.1186/s12885-017-3948-3 (PMC5763897; doi:10.1186/s12885-017-3948-3)
Supplement: Supplementary file 6 — Fusion genes selected by customized filtering steps and experimentally validated by RT-PCR and Sanger sequencing. FTC, follicular thyroid carcinoma; ORF, open reading frame. (DOCX 15 kb) [file 12885_2017_3948_MOESM6_ESM.docx]

**Supplementary table 3** Fusion genes selected by customized filtering steps and experimentally validated by RT-PCR and Sanger sequencing. FTC, follicular thyroid carcinoma; ORF, open reading frame.

| Fusion gene^1^ | Upstream gene | | Downstream gene | | Distance (kb)^2^ | ORF | FTC sample |
| --- | --- | --- | --- | --- | --- | --- | --- |
|  | Chr.^3^ | Gene ID | Chr.^3^ | Gene ID |  |  |  |
| Interchromosomal |  |  |  |  |  |  |  |
| *DISP1-SUPT20H* | 1q41 | ENSG00000154309 | 13q13.3 | ENSG00000102710 | - | No | Case 2, 4 |
| *EML2-C16orf46* | 19q13.32 | ENSG00000125746 | 16q23.2 | ENSG00000166455 | - | No | Case 1 |
| *FBXO25-BET1L*^4^ | 8p23.3 | ENSG00000147364 | 11p15.5 | ENSG00000177951 | - | Yes | Case 4 |
| *FBXO25-RP11-261C10.3*^4^ | 8p23.3 | ENSG00000147364 | 1q43 | ENSG00000214837 | - | No | Case 2 |
| *GSN-KIAA0586* | 9q33.2 | ENSG00000148180 | 14q23.1 | ENSG00000100578 | - | No | Case 2, 4 |
| *HIBCH-ERI2* | 2q32.2 | ENSG00000198130 | 16p12.3 | ENSG00000196678 | - | No | Case 2, 4 |
| *NUBPL-PPP1R3F* | 14q12 | ENSG00000151413 | Xp11.23 | ENSG00000049769 | - | No | Case 3 |
| *PHKA2-SYTL3* | Xp22.13 | ENSG00000044446 | 6q25.3 | ENSG00000164674 | - | No | Case 4 |
| *PPP1R3F-NUBPL* | Xp11.23 | ENSG00000049769 | 14q12 | ENSG00000151413 | - | No | Case 1, 2, 3 |
| *RBM27-FCGBP* | 5q32 | ENSG00000091009 | 19q13.2 | ENSG00000090920 | - | No | Case 2 |
| *SAV1-GYPE* | 14q22.1 | ENSG00000151748 | 4q31.21 | ENSG00000197465 | - | Yes | Case 3 |
| *SCRN3-SCFD1* | 2q31.1 | ENSG00000144306 | 14q12 | ENSG00000092108 | - | No | Case 4 |
| *SCRN3-RABGAP1L* | 2q31.1 | ENSG00000144306 | 1q25.1 | ENSG00000152061 | - | No | Case 2 |
| *SLC22A20-PPARD* | 11q13.1 | ENSG00000197847 | 6p21.31 | ENSG00000112033 | - | No | Case 3 |
|  |  |  |  |  |  |  |  |
| Intrachromosomal |  |  |  |  |  |  |  |
| *C1orf196-KAZN* | 1p36.21 | ENSG00000218328 | 1p36.21 | ENSG00000189337 | 706 | Yes | Case 1 |
| *KIAA1267-ARL17A*^5^ | 17q21.31 | ENSG00000120071 | 17q21.31 | ENSG00000185829 | 487 | Yes | Case 1 |
| *KIAA1267-ARL17B*^5^ | 17q21.31 | ENSG00000120071 | 17q21.31 | ENSG00000228696 | 269 | No | Case 4 |
| *LOC728613-SDHA* | 5p15.33 | ENSG00000188002 | 5p15.33 | ENSG00000073578 | 138 | No | Case 4 |
| *MIR4435-1HG-ANAPC1* | 2q13 | ENSG00000172965 | 2q13 | ENSG00000153107 | 558 | No | Case 3, 4 |
| *RP11-141M1.4-STARD13* | 13q13.1 | ENSG00000233001 | 13q13.1 | ENSG00000133121 | 245 | No | Case 2, 3, 4 |
| *RP11-634B7.4-TRIM58* | 1q44 | ENSG00000235749 | 1q44 | ENSG00000162722 | 217 | No | Case 1 |
|  |  |  |  |  |  |  |  |

1, all fusion genes were experimentally validated for all the four follicular thyroid cancers and their corresponding adjacent normal thyroid tissues; 2, distance is the outer distance between the two genes; 3, Ensembl cytogenetic band information; 4, *RP11-216C10.3* and *BET1L* are homologous; 5, *ARL17A* and *ARL17B* are homologous.
