# Supplementary material for: CRABP1, C1QL1 and LCN2 are biomarkers of differentiated thyroid carcinoma, and predict extrathyroidal extension
Source: BMC Cancer. 2018 Jan 10;18:68. doi: 10.1186/s12885-017-3948-3 (PMC5763897; doi:10.1186/s12885-017-3948-3)
Supplement: Supplementary file 7 — Oligonucleotide primers used in RT-PCR to detect the fusion genes. (DOCX 13 kb) [file 12885_2017_3948_MOESM7_ESM.docx]

**Supplementary table 4** Oligonucleotide primers used in RT-PCR to detect the fusion genes.

|  | Fusion gene | Forward primer | Reverse primer |
| --- | --- | --- | --- |
|  | *DISP1-SUPT20H* | GTACCAGCTCCTCCTTGTGC | TGGCATAAAATAAGGGTCCAA |
|  | *EML2-C16orf46* | CCCGAGTAGCTGGGATTACA | AAGACCTTGCTCCAAACGTG |
|  | *FBXO25-BET1L* | GCTATTGCACCTTGGGAGAA | GAGGGACAGAGGCAGATGAG |
|  | *FBXO25-RP11-261C10.3* | GCTATTGCACCTTGGGAGAA | GAGGGACAGAGGCAGATGAG |
|  | *GSN-KIAA0586* | TGCCTTTTGGAACTGTCCTT | TGAGAATTATGAAGCCACTTACTTG |
|  | *HIBCH-ERI2* | GCAAAGGCAGAGGAGAACAA | ATTTCCTGGCTATGGTGGTG |
|  | *NUBPL-PPP1R3F* | CCTGTTTGCCTGGGTATCAC | AGCCTGCATCATTTCAGCTT |
|  | *PHKA2-SYTL3* | AGGCTGGTCTTGAACTCCTG | CAGGAGGTGGAGTAGGAGGA |
|  | *PPP1R3F-NUBPL* | TGATGTCCAGGAGTCAGTGG | GAAGGAACAGGCAGCAATCT |
|  | *RBM27-FCGBP* | AGTGATTCTCCTGCCTCAGC | GTAGTTTCCAGGGACGGTGA |
|  | *SAV1-GYPE* | TTCCAAAGAATGCCACAGAA | TCATTCCAACAACAACAAGCA |
|  | *SCRN3-SCFD1* | TTTAAGCCGGTCTGAAAAGC | GAAACAACCTCATTTTATCTTCTGG |
|  | *SCRN3-RABGAP1L* | TTTAAGCCGGTCTGAAAAGC | CGAACCATGGGAACTCTCTG |
|  | *SLC22A20-PPARD* | AGACCCTCTGCCTGGCAAC | ATAGCTCTGGCATCGTCTGG |
|  | *C1orf196-KAZN* | GCACACCCTCAATGACCAG | TGTCTTCCATCATGCTCAGG |
|  | *KIAA1267-ARL17A* | ACTGGCTTCAGGCTCATGTT | AAAATGCTGCCACAGAGGTC |
|  | *KIAA1267-ARL17B* | ACTGGCTTCAGGCTCATGTT | AAAATGCTGCCACAGAGGTC |
|  | *LOC728613-SDHA* | CGTGGACTCCCTTTAATCCA | AGACAACCAGGTCCAAGAGC |
|  | *MIR4435-1HG-ANAPC1* | GACAATGATAAAGGGCTCTGAAA | TCAGATCCAGTCTCGGAAGG |
|  | *RP11-141M1.4-STARD13* | AATGACCAGCAAACGGAAAC | CACATGCTTCTTTTGCCTCA |
|  | *RP11-634B7.4-TRIM58* | CGAACATCTCCAGGATGTGA | AAGGCGTCCTCCAACTCTTT |
|  |  |  |  |
