# Supplementary material for: CRABP1, C1QL1 and LCN2 are biomarkers of differentiated thyroid carcinoma, and predict extrathyroidal extension
Source: BMC Cancer. 2018 Jan 10;18:68. doi: 10.1186/s12885-017-3948-3 (PMC5763897; doi:10.1186/s12885-017-3948-3)
Supplement: Supplementary file 8 — Prediction and experimental validation of the fusion genes expressed in follicular thyroid carcinomas used in RNA-sequencing. (DOCX 17 kb) [file 12885_2017_3948_MOESM8_ESM.docx]

**Prediction and experimental validation of the fusion genes expressed in follicular thyroid carcinomas (FTC) used in RNA-sequencing**

**Methods**

**Fusion gene predictions**

A particular attention was paid to the identification of novel fusion transcripts between minimally and widely invasive FTC. Reads marked by the Illumina pipeline (Bustard.py, OLB 1.6.0 and 1.8.0) as *passed filtering* were used in the analysis. Survey of potential fusion genes in FTCs was performed by the deFuse software [[1](#_ENREF_1)], following the procedure used in Nome et al. [[2](#_ENREF_2)]. Briefly, each fusion was required to be supported by at least three split sequence reads harbouring the fusion breakpoint sequence, and two spanning read pairs mapping discordantly to one of the partner genes each. Further, customized filtering steps of the fusions were performed where the first step included removing fusions where the fusion breakpoint occurred in intronic or intraexonic sequences of the partner genes, leaving only fusion transcripts with intact annotated exon-exon boundaries, using predominantly consensus splice sites. The second step removed intrachromosomal fusions where the gene partners were located less than 100 kbp apart. The third step removed fusions where one of the genes had multiple partners within the same FTC sample. The last step removed fusions with partner homology.

**Experimental validation of nominated fusion genes**

For nominated fusion genes, a forward primer was made to anneal in the last exon upstream of the breakpoint and a reverse primer in the first exon downstream of the breakpoint (Additional file 7: Table S4). The PCR using cDNA was performed with HotStar Taq DNA polymerase kit (Qiagen Co., Valencia, CA, USA), and initiated by denaturation at 95°C for 15 minutes, followed by 30 cycles of 30 seconds at 95°C (denaturation), 75 seconds at 56-59°C (annealing), and 30 seconds at 72°C (elongation), before a final elongation step at 72°C for 6 minutes. The RT-PCR products were analysed by 2 % agarose gel electrophoresis. PCR reactions creating a visible band on the gel were sequenced directly by Sanger sequencing.

**Results**

From a total of 245 million pair-end reads, the software tool deFuse identified 6439 fusion transcript candidates in the FTC (Additional file 5: Figure S2). We selected 29 fusion transcripts by stringent requirements. In this customized filtering, the first step removed fusion transcripts where the fusion breakpoint occurred in intronic or intraexonic sequences of the partner genes, leaving only fusion transcripts with intact annotated exon-exon boundaries, resulting in 162 fusion transcripts. The second step removed intrachromosomal fusions, where the gene partners were located less than 100 kbp apart, leaving 82 fusion transcripts. The third step removed fusions where one of the genes had multiple partners within the same FTC sample, leaving 68 fusion genes. The last step removed fusions with partner homology, resulting in a final 29 candidate fusion transcripts. Of the 29 candidate fusion transcripts there were 21 unique fusion breakpoints and six of these were recurrent in two or more FTC samples (Additional file 6: Table S3). All these 21 fusion genes were experimentally validated by reverse-transcription PCR and cDNA Sanger sequencing in the corresponding FTC and in its matched normal tissue.

# References

1. McPherson A, Hormozdiari F, Zayed A, Giuliany R, Ha G, Sun MG, Griffith M, Heravi Moussavi A, Senz J, Melnyk N, et al. deFuse: an algorithm for gene fusion discovery in tumor RNA-Seq data. PLoS Comput Biol. 2011; 7:e1001138.

2. Nome T, Thomassen GO, Bruun J, Ahlquist T, Bakken AC, Hoff AM, Rognum T, Nesbakken A, Lorenz S, Sun J, et al. Common fusion transcripts identified in colorectal cancer cell lines by high-throughput RNA sequencing. Transl Oncol. 2013; 6:546-553.
