# Supplementary material for: CRABP1, C1QL1 and LCN2 are biomarkers of differentiated thyroid carcinoma, and predict extrathyroidal extension
Source: BMC Cancer. 2018 Jan 10;18:68. doi: 10.1186/s12885-017-3948-3 (PMC5763897; doi:10.1186/s12885-017-3948-3)
Supplement: Supplementary file 10 — Receiver Operating Characteristics (ROC) curve analysis. ROC curves for individual biomarkers were generated using log2 (2-ΔΔCT) gene expression values and thyroid tissue type [differentiated thyroid carcinoma (DTC) and normal or DTC and follicular thyroid adenoma (FTA)] as input. For evaluation of the combined biomarker panel the sum of log2 (2-ΔΔCT) expression values from genes with gain (C1QL1 and LCN2) and loss (CRABP1 and CILP) in DTC were used. AUC, area under the curve; CI, confidence interval. (DOCX 14 kb) [file 12885_2017_3948_MOESM10_ESM.docx]

**Supplementary table 5** Receiver Operating Characteristics (ROC) curve analysis. ROC curves for individual biomarkers were generated using log2 (2^-ΔΔCT^) gene expression values and thyroid tissue type [differentiated thyroid carcinoma (DTC) and normal or DTC and follicular thyroid adenoma (FTA)] as input. For evaluation of the combined biomarker panel the sum of log2 (2^-ΔΔCT^) expression values from genes with gain (*C1QL1* and *LCN2*) and loss (*CRABP1* and *CILP*) in DTC were used. AUC, area under the curve; CI, confidence interval.

|  | Biomarkers | AUC | Standard error | Asymptotic significance | 95% CI lower | 95% CI upper |
| --- | --- | --- | --- | --- | --- | --- |
| DTC Vs Normal | *C1QL1* | 0.799 | 0.053 | 2.1E-3 | 0.694 | 0.903 |
|  | *LCN2* | 0.783 | 0.061 | 3.6E-3 | 0.664 | 0.902 |
|  | *CRABP1* | 0.902 | 0.033 | 3.4E-5 | 0.838 | 0.966 |
|  | *CILP* | 0.687 | 0.056 | 5.5E-2 | 0.576 | 0.797 |
|  | Panel | 0.927 | 0.027 | 1.1E-5 | 0.874 | 0.979 |
| DTC Vs FTA | *C1QL1* | 0.566 | 0.081 | 4.8E-1 | 0.408 | 0.725 |
|  | *LCN2* | 0.898 | 0.040 | 1.9E-5 | 0.819 | 0.977 |
|  | *CRABP1* | 0.746 | 0.064 | 8.3E-3 | 0.621 | 0.871 |
|  | *CILP* | 0.675 | 0.080 | 6.1E-2 | 0.518 | 0.831 |
|  | Panel | 0.839 | 0.051 | 2.7E-4 | 0.740 | 0.938 |
