# Supplementary material for: CRABP1, C1QL1 and LCN2 are biomarkers of differentiated thyroid carcinoma, and predict extrathyroidal extension
Source: BMC Cancer. 2018 Jan 10;18:68. doi: 10.1186/s12885-017-3948-3 (PMC5763897; doi:10.1186/s12885-017-3948-3)
Supplement: Supplementary file 12 — Clinicopathological and genetic data of the FTC classified by classes based on gene expression. (DOCX 18 kb) [file 12885_2017_3948_MOESM12_ESM.docx]

**Supplementary table 6** Clinicopathological and genetic data of the FTC classified by classes based on gene expression.

|  | *C1QL1* fold change | |  | *LCN2* fold change | |  | *CRABP1* fold change | |  | *CILP* fold change | |  |
| --- | --- | --- | --- | --- | --- | --- | --- | --- | --- | --- | --- | --- |
|  | **Normal ≤1** | **Gain >1** | ***P* value** | **Normal ≤1** | **Gain >1** | ***P* value** | **Loss <-1** | **Normal ≥-1** | ***P* value** | **Loss <-1** | **Normal ≥-1** | ***P* value** |
| FTC (n=15) |  |  |  |  |  |  |  |  |  |  |  |  |
| Age (n)  Mean (years) | 8  49.8±4.8 | 7  55.9±6.3 | NS (0.463) | 8  47.75±3.5 | 6  63.0±5.9 | NS (0.059) | 13  52.9±4.5 | 2  51.0±2.0 | NS (0.933) | 9  55.8±5.7 | 5  51.6±2.3 | NS (0.898) |
| Tumour size (n)  Mean (cm) | 8  3.34±0.42 | 7  4.61±0.84 | NS (0.232) | 8  4.03±0.67 | 6  3.30±0.53 | NS (0.491) | 13  3.81±0.52 | 2  4.75±0.75 | NS (0.381) | 9  3.71±0.61 | 5  3.72±0.66 | NS (1.000) |
| Gender (n)  Female (%)  Male (%) | 8  6 (75.0)  2 (25.0) | 7  6 (85.7)  1 (14.3) | NS (0.554) | 8  7 (87.5)  1 (12.5) | 6  4 (66.7)  2 (33.3) | NS (0.385) | 13  10 (76.9)  3 (23.1) | 2  2 (100)  - | NS (0.629) | 9  6 (66.7)  3 (33.3) | 5  5 (100)  - | NS (0.231) |
| Capsule (n)  Positive (%) | 8  8 (100) | 7  7 (100) | ^1^ | 8  8 (100) | 6  (100) | ^1^ | 13  13 (100) | 2  2 (100) | ^1^ | 9  9 (100) | 5  5 (100) | ^1^ |
| Capsular invasion (n)  Positive (%) | 8  8 (100) | 7  7 (100) | ^1^ | 8  8 (100) | 6  (100) | ^1^ | 13  13 (100) | 2  2 (100) | ^1^ | 9  9 (100) | 5  5 (100) | ^1^ |
| Invasiveness degree (n)  Minimally invasive (%)  Widely invasive (%) | 8  8 (100)  - | 7  2 (28.6)  5 (71.4) | **0.007** | 8  5 (62.5)  3 (37.5) | 6  4 (66.7)  2 (33.3) | NS (0.657) | 13  9 (69.2)  4 (30.8) | 2  1 (50.0)  1 (50.0) | NS (0.571) | 9  5 (55.6)  4 (44.4) | 5  4 (80.0)  1 (20.0) | NS (0.378) |
| Vascular invasion (n)  Positive (%) | 8  6 (75.0) | 7  5 (71.4) | NS (0.662) | 8  5 (62.5) | 6  5 (83.3) | NS (0.406) | 13  10 (76.9) | 2  1 (50.0) | NS (0.476) | 9  6 (66.7) | 5  4 (80.0) | NS (0.545) |
| Lymph node metastasis (n)  Positive (%) | 8  - | 7  - | ^1^ | 8  - | 6  - | ^1^ | 13  - | 2  - | ^1^ | 9  - | 5  - | ^1^ |
| Extrathyroidal extension (n)  Positive (%) | 8  - | 7  1 (14.3) | NS (0.467) | 8  1 (12.5) | 6  - | NS (0.571) | 13  1 (7.69) | 2  - | NS (0.867) | 9  1 (11.1) | 5  - | NS (0.643) |
| Distant metastasis (n)  Positive (%) | 8  - | 7  1 (14.3) | NS (0.467) | 8  1 (12.5) | 6  - | NS (0.571) | 13  1 (7.69) | 2  - | NS (0.867) | 9  1 (11.1) | 5  - | NS (0.643) |
| Lymphocytic thyroiditis (n)  Positive (%) | 8  - | 7  4 (57.1) | **0.026** | 8  1 (12.5) | 6  2 (33.3) | NS (0.385) | 13  3 (23.1) | 2  1 (50.0) | NS (0.476) | 9  2 (22.2) | 5  1 (20.0) | NS (0.725) |
| Oncocytic (n)  Positive (%) | 8  2 (25.0) | 7  4 (57.1) | NS (0.231) | 8  1 (12.5) | 6  5 (83.3) | **0.016** | 13  5 (38.4) | 2  1 (50.0) | NS (0.657) | 9  5 (55.6) | 5  1 (20.0) | NS (0.238) |
| *PAX8-PPARG* rearrangements (n)  Positive (%) | 8  1 (12.5) | 7  - | NS (0.533) | 8  1 (12.5) | 6  - | NS (0.571) | 13  1 (7.69) | 2  - | NS (0.867) | 9  - | 5  1 (20.0) | NS (0.357) |
| *RET/PTC* rearrangements (n)  Positive (%) | 8  1 (12.5) | 7  - | NS (0.533) | 8  1 (12.5) | 6  - | NS (0.571) | 13  1 (7.69) | 2  - | NS (0.867) | 9  - | 5  1 (20.0) | NS (0.357) |
| *RET/PTC1* rearrangement (n)  Positive (%) | 8  1 (12.5) | 7  - | NS (0.533) | 8  1 (12.5) | 6  - | NS (0.571) | 13  1 (7.69) | 2  - | NS (0.867) | 9  - | 5  1 (20.0) | NS (0.357) |
| *RET/PTC3* rearrangement (n)  Positive (%) | 8  - | 7  - | ^1^ | 8  - | 6  - | ^1^ | 13  - | 2  - | ^1^ | 9  - | 5  - | ^1^ |
| *BRAF* mutation (n)  Positive (%) | 8  - | 7  - | ^1^ | 8  - | 6  - | ^1^ | 13  - | 2  - | ^1^ | 9  - | 5  - | ^1^ |
| *NRAS* mutation (n)  Positive (%) | 8  1 (12.5) | 7  1 (14.3) | NS (0.100) | 8  3 (37.5) | 6  2 (33.3) | NS (0.657) | 13  5 (38.5) | 2  - | NS (0.429) | 9  5 (55.6) | 5  - | NS (0.063) |
| *TERT* promoter mutation (n)  Positive (%) | 8  - | 7  2 (28.6) | NS (0.200) | 8  1 (12.5) | 6  1 (16.7) | NS (0.692) | 13  2 (15.4) | 2  - | NS (0.743) | 9  2 (22.2) | 5  - | NS (0.396) |
|  |  |  |  |  |  |  |  |  |  |  |  |  |

n, number of cases with available data; 1, no statistics were computed due to constant numbers of one feature.
