# Supplementary material for: CRABP1, C1QL1 and LCN2 are biomarkers of differentiated thyroid carcinoma, and predict extrathyroidal extension
Source: BMC Cancer. 2018 Jan 10;18:68. doi: 10.1186/s12885-017-3948-3 (PMC5763897; doi:10.1186/s12885-017-3948-3)
Supplement: Supplementary file 13 — Clinicopathological and genetic data of the FVPTC classified by classes based on gene expression. (DOCX 17 kb) [file 12885_2017_3948_MOESM13_ESM.docx]

**Supplementary table 7** Clinicopathological and genetic data of the FVPTC classified by classes based on gene expression.

|  | *C1QL1* fold change | |  | *LCN2* fold change | |  | *CRABP1* fold change | |  | *CILP* fold change | |  |
| --- | --- | --- | --- | --- | --- | --- | --- | --- | --- | --- | --- | --- |
|  | **Normal ≤1** | **Gain >1** | ***P* value** | **Normal ≤1** | **Gain >1** | ***P* value** | **Loss <-1** | **Normal ≥-1** | ***P* value** | **Loss <-1** | **Normal ≥-1** | ***P* value** |
| FVPTC (n=23) |  |  |  |  |  |  |  |  |  |  |  |  |
| Age (n)  Mean (years) | 8  36.5±3.17 | 12  46.4±4.67 | NS (0.115) | 8  39.5±2.9 | 11  45.2±5.4 | NS (0.310) | 14  43.1±4.0 | 5  41.20±7.1 | NS (1.000) | 10  37.2±3.5 | 10  47.7±5.0 | NS (0.105) |
| Tumour size (n)  Mean (cm) | 7  1.87±0.43 | 12  2.58±0.44 | NS (0.432) | 8  2.51±0.55 | 10  2.09±0.45 | NS (0.573) | 13  2.58±0.42 | 5  1.84±0.52 | NS (0.556) | 9  1.72±0.33 | 10  2.85±0.50 | NS (0.095) |
| Gender (n)  Female (%)  Male (%) | 8  6 (75.0)  2 (25.0) | 12  10 (83.3)  2 (16.7) | NS (0.535) | 8  7 (87.5)  1 (12.5) | 11  8 (72.7)  3 (27.3) | NS (0.426) | 14  11 (78.6)  3 (21.4) | 5  4 (80.0)  1 (20.0) | NS (0.728) | 10  8 (80.0)  2 (20.0) | 10  8 (80.0)  2 (20.0) | NS (0.709) |
| Capsule (n)  Positive (%) | 8  2 (25.0) | 10  6 (60.0) | NS (0.157) | 7  5 (71.4) | 10  3 (30.0) | NS (0.117) | 12  7 (58.3) | 5  1 (20.0) | NS (0.183) | 5  4 (80.0) | 5  4 (80.0) | NS (0.681) |
| Capsular invasion (n)  Positive (%) | 2  - | 5  2 (40.0) | NS (0.476) | 4  1 (25.0) | 3  1 (33.3) | NS (0.714) | 6  2 (33.3) | 1  - | NS (0.714) | 4  - | 3  2 (66.7) | NS (0.143) |
| Vascular invasion (n)  Positive (%) | 7  3 (42.9) | 11  2 (18.2) | NS (0.272) | 8  3 (37.5) | 9  2 (22.2) | NS (0.437) | 12  5 (41.7) | 5  - | NS (0.128) | 9  3 (33.3) | 9  2 (22.2) | NS (0.500) |
| Lymph node metastasis (n)  Positive (%) | 7  1 (14.3) | 11  3 (27.3) | NS (0.485) | 8  1 (12.5) | 9  3 (33.3) | NS (0.335) | 12  4 (33.3) | 5  - | NS (0.208) | 9  2 (22.2) | 9  2 (22.2) | NS (0.712) |
| Extrathyroidal extension (n)  Positive (%) | 7  1 (14.3) | 11  1 (9.09) | NS (0.641) | 8  - | 9  2 (22.2) | NS (0.265) | 12  2 (16.7) | 5  - | NS (0.485) | 9  1 (11.1) | 9  1 (11.1) | NS (0.765) |
| Distant metastasis (n)  Positive (%) | 7  - | 11  - | ^1^ | 8  - | 9  - | ^1^ | 12  - | 5  - | ^1^ | 9  - | 9  - | ^1^ |
| Lymphocytic thyroiditis (n)  Positive (%) | 7  1 (14.3) | 12  6 (50.0) | NS (0.144) | 8  4 (50.0) | 10  3 (30.0) | NS (0.352) | 13  6 (46.2) | 5  1 (20.0) | NS (0.324) | 9  3 (33.3) | 10  4 (40.0) | NS (0.570) |
| Oncocytic (n)  Positive (%) | 7  - | 11  - | ^1^ | 8  - | 9  - | ^1^ | 12  - | 5  - | ^1^ | 9  - | 9  - | ^1^ |
| *PAX8-PPARG* rearrangements (n)  Positive (%) | 8  - | 12  - | ^1^ | 8  - | 11  - | ^1^ | 14  - | 5  - | ^1^ | 10  - | 10  - | ^1^ |
| *RET/PTC* rearrangements (n)  Positive (%) | 8  - | 12  2 (16.7) | NS (0.347) | 8  - | 11  2 (18.2) | NS (0.322) | 14  1 (7.14) | 5  1 (20.0) | NS (0.468) | 10  - | 10  2 (20.0) | NS (0.237) |
| *RET/PTC1* rearrangement (n)  Positive (%) | 8  - | 12  1 (8.33) | NS (0.600) | 8  - | 11  1 (9.09) | NS (0.579) | 14  1 (7.14) | 5  - | NS (0.737) | 10  - | 10  1 (10.0) | NS (0.500) |
| *RET/PTC3* rearrangement (n)  Positive (%) | 8  - | 12  - | ^1^ | 8  - | 11  - | ^1^ | 14  - | 5  - | ^1^ | 10  - | 10  - | ^1^ |
| *BRAF* mutation (n)  Positive (%) | 8  - | 12  2 (16.7) | NS (0.347) | 8  - | 11  2 (18.2) | NS (0.322) | 14  2 (14.3) | 5  - | NS (0.532) | 10  1 (10.0) | 10  1 (10.0) | NS (0.763) |
| *NRAS* mutation (n)  Positive (%) | 8  4 (50.0) | 12  - | **0.014** | 8  3 (37.5) | 11  1 (9.09) | NS (0.177) | 14  3 (21.4) | 5  - | NS (0.376) | 10  4 (40.0) | 10  - | **0.043** |
| *TERT* promoter mutation (n)  Positive (%) | 8  - | 12  1 (8.33) | NS (0.600) | 8  - | 11  1 (9.09) | NS (0.579) | 14  1 (7.14) | 5  - | NS (0.737) | 10  - | 10  1 (10.0) | NS (0.500) |

n, number of cases with available data; 1, no statistics were computed due to constant numbers of one feature.
