# Supplementary material for: CRABP1, C1QL1 and LCN2 are biomarkers of differentiated thyroid carcinoma, and predict extrathyroidal extension
Source: BMC Cancer. 2018 Jan 10;18:68. doi: 10.1186/s12885-017-3948-3 (PMC5763897; doi:10.1186/s12885-017-3948-3)
Supplement: Supplementary file 14 — Clinicopathological and genetic data of the PTC classified by classes based on gene expression. (DOCX 17 kb) [file 12885_2017_3948_MOESM14_ESM.docx]

**Supplementary table 8** Clinicopathological and genetic data of the PTC classified by classes based on gene expression.

|  | *C1QL1* fold change | |  | *LCN2* fold change | |  | *CRABP1* fold change | |  | *CILP* fold change | |  |
| --- | --- | --- | --- | --- | --- | --- | --- | --- | --- | --- | --- | --- |
|  | **Normal ≤1** | **Gain >1** | ***P* value** | **Normal ≤1** | **Gain >1** | ***P* value** | **Loss <-1** | **Normal ≥-1** | ***P* value** | **Loss <-1** | **Normal ≥-1** | ***P* value** |
| PTC (n=60) |  |  |  |  |  |  |  |  |  |  |  |  |
| Age (n)  Mean (years) | 17  38.8±3.5 | 37  41.3±2.9 | NS (0.608) | 15  40.2±3.7 | 38  40.58±2.7 | NS (0.961) | 48  40.9±2.4 | 7  39.29±5.5 | NS (0.814) | 35  41.3±2.8 | 19  40.0±3.8 | NS (0.928) |
| Tumour size (n)  Mean (cm) | 16  1.78±0.25 | 35  2.96±0.33 | **0.021** | 13  1.76±0.34 | 37  2.72±0.30 | **0.023** | 45  2.67±0.27 | 7  1.91±0.54 | NS (0.184) | 33  2.95±0.37 | 18  1.95±0.19 | NS (0.150) |
| Gender (n)  Female (%)  Male (%) | 17  14 (82.4)  3 (17.6) | 37  34 (91.9)  3 (8.1) | NS (0.275) | 15  14 (93.3)  1 (6.7) | 38  33 (86.8)  5 (13.2) | NS (0.448) | 48  42 (87.5)  6 (12.5) | 7  7 (100)  - | NS (0.423) | 35  30 (85.7)  5 (14.3) | 19  18 (94.7)  1 (5.3) | NS (0.302) |
| Capsule (n)  Positive (%) | 14  8 (57.1) | 33  12 (36.4) | NS (0.160) | 11  4 (36.4) | 35  15 (42.9) | NS (0.492) | 41  19 (46.3) | 7  1 (14.3) | NS (0.118) | 31  16 (51.6) | 16  4 (25.0) | NS (0.074) |
| Capsular invasion (n)  Positive (%) | 7  4 (57.1) | 11  6 (54.5) | NS (0.648) | 3  2 (66.7) | 14  7 (50.0) | NS (0.547) | 18  10 (55.6) | - | ^1^ | 15  8 (53.3) | 3  2 (66.7) | NS (0.588) |
| Vascular invasion (n)  Positive (%) | 15  8 (53.3) | 33  23 (69.7) | NS (0.219) | 12  6 (50.0) | 36  24 (66.7) | NS (0.244) | 42  27 (64.3) | 7  4 (57.1) | NS (0.512) | 32  21 (65.6) | 16  9 (56.3) | NS (0.373) |
| Lymph node metastasis (n)  Positive | 16  6 (37.5) | 36  12 (33.3) | NS (0.504) | 14  4 (28.6) | 38  15 (39.5) | NS (0.350) | 46  17 (37.0) | 7  3 (42.9) | NS (0.536) | 33  8 (24.2) | 19  10 (52.6) | NS (0.039) |
| Extrathyroidal extension (n)  Positive (%) | 15  3 (20.0) | 33  21 (63.6) | **0.006** | 13  4 (30.8) | 35  19 (54.3) | NS (0.130) | 42  20 (47.6) | 6  3 (50.0) | NS (0.625) | 30  15 (50.0) | 18  8 (44.4) | NS (0.471) |
| Distant metastasis (n)  Positive (%) | 16  - | 36  4 (11.1) | NS (0.218) | 14  - | 38  4 (10.5) | NS (0.273) | 46  4 (8.70) | 7  - | NS (0.557) | 33  2 86.06) | 19  2 (10.5) | NS (0.466) |
| Lymphocytic thyroiditis (n)  Positive (%) | 17  6 (35.3) | 32  18 (56.3) | NS (0.136) | 14  7 (50.0) | 35  18 (51.4) | NS (0.589) | 43  22 (51.2) | 7  4 (57.1) | NS (0.547) | 31  14 (45.2) | 18  10 (55.6) | NS (0.343) |
| Oncocytic (n)  Positive (%) | 16  3 (18.8) | 32  8 (25.0) | NS (0.461) | 12  1 (8.33) | 36  10 (27.8) | NS (0.162) | 42  8 (19.0) | 7  3 (42.9) | NS (0.178) | 30  4 (13.3) | 18  6 (33.3) | NS (0.101) |
| *PAX8-PPARG* rearrangements (n)  Positive (%) | 17  - | 37  - | ^1^ | 15  - | 38  - | ^1^ | 48  - | 7  - | ^1^ | 35  - | 19  - | ^1^ |
| *RET/PTC* rearrangements (n)  Positive (%) | 17  4 (23.5) | 37  8 (21.6) | NS (0.567) | 15  2 (13.3) | 38  9 (23.7) | NS (0.333) | 48  10 (20.8) | 7  1 (14.3) | NS (0.571) | 35  7 (20.0) | 19  5 (26.3) | NS (0.418) |
| *RET/PTC1* rearrangement (n)  Positive (%) | 17  3 (17.6) | 37  6 (16.2) | NS (0.590) | 15  1 (6.67) | 38  8 (21.1) | NS (0.202) | 48  10 (20.8) | 7  1 (14.3) | NS (0.679) | 35  4 (11.4) | 19  5 (26.3) | NS (0.154) |
| *RET/PTC3* rearrangement (n)  Positive (%) | 17  - | 37  1 (2.70) | NS (0.685) | 15  - | 38  - | ^1^ | 48  - | 7  - | ^1^ | 35  1 (2.86) | 19  - | NS (0.648) |
| *BRAF* mutation (n)  Positive (%) | 17  - | 37  16 (43.2) | **0.001** | 15  3 (20.0) | 38  14 (36.8) | NS (0.198) | 48  17 (35.4) | 7  - | NS (0.062) | 35  12 (34.3) | 19  5 (26.3) | NS (0.388) |
| *NRAS* mutation (n)  Positive (%) | 17  2 (11.8) | 37  3 (8.11) | NS (0.507) | 15  - | 38  4 (10.5) | NS (0.252) | 48  5 (10.4) | 7  - | NS (0.492) | 35  5 (14.3) | 19  - | NS (0.103) |
| *TERT* promoter mutation (n)  Positive (%) | 17  - | 37  - | ^1^ | 15  - | 38  - | ^1^ | 48  - | 7  - | ^1^ | 35  - | 19  - | ^1^ |

n, number of cases with available data; 1, no statistics were computed due to constant numbers of one feature
